# A metabolically stable apelin-17 analog decreases AVP-induced antidiuresis and improves hyponatremia

**DOI:** 10.1038/s41467-020-20560-y

**Published:** 2021-01-12

**Authors:** Adrien Flahault, Pierre-Emmanuel Girault-Sotias, Mathilde Keck, Rodrigo Alvear-Perez, Nadia De Mota, Lucie Estéoulle, Sridévi M. Ramanoudjame, Xavier Iturrioz, Dominique Bonnet, Catherine Llorens-Cortes

**Affiliations:** 1 Laboratory of Central Neuropeptides in the Regulation of Body Fluid Homeostasis and Cardiovascular Functions, Center for Interdisciplinary Research in Biology, INSERM, Unit U1050, Centre National de la Recherche Scientifique, Unite Mixte de Recherche 7241, Collège de France, Paris, France; 2 Laboratory of Therapeutic Innovation, Unité Mixte de Recherche 7200, Centre National de la Recherche Scientifique, Faculty of Pharmacy, University of Strasbourg, Illkirch, France

**Keywords:** Biochemistry, Peptides, Endocrinology, Nephrology

## Abstract

Apelin and arginine-vasopressin (AVP) are conversely regulated by osmotic stimuli. We therefore hypothesized that activating the apelin receptor (apelin-R) with LIT01-196, a metabolically stable apelin-17 analog, may be beneficial for treating the Syndrome of Inappropriate Antidiuresis, in which AVP hypersecretion leads to hyponatremia. We show that LIT01-196, which behaves as a potent full agonist for the apelin-R, has an in vivo half-life of 156 minutes in the bloodstream after subcutaneous administration in control rats. In collecting ducts, LIT01-196 decreases dDAVP-induced cAMP production and apical cell surface expression of phosphorylated aquaporin 2 via AVP type 2 receptors, leading to an increase in aqueous diuresis. In a rat experimental model of AVP-induced hyponatremia, LIT01-196 subcutaneously administered blocks the antidiuretic effect of AVP and the AVP-induced increase in urinary osmolality and induces a progressive improvement of hyponatremia. Our data suggest that apelin-R activation constitutes an original approach for hyponatremia treatment.

## Abstract (web summary)

Apelin and AVP have opposing effects water balance in humans and rodents. Here, the authors report that a metabolically stable apelin-17 analog, by acting at the kidney level, reduces AVP-induced antidiuresis and improves hyponatremia in rodents, demonstrating a potential approach for treating water metabolism disorders.

## Introduction

Hyponatremia, defined in humans as a plasma sodium concentration below 135 mmol/l, is associated with various diseases, including chronic heart failure (CHF), liver cirrhosis, diuretic treatment, and the syndrome of inappropriate antidiuresis (SIAD)^1^. Hyponatremia is also associated with high mortality rates^2^ in patients with liver failure^3^, CHF^4^, and chronic kidney disease^5^.

**Fig. 1:**
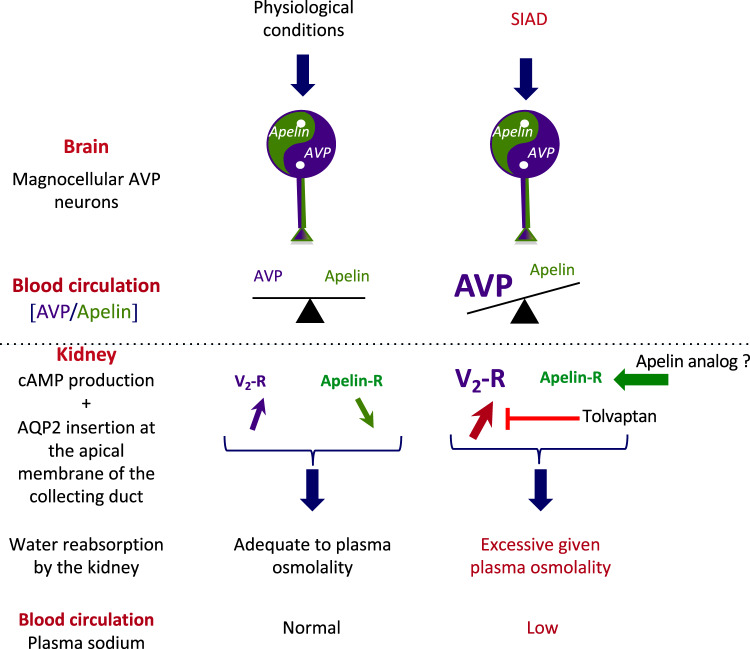
Apelin and vasopressin regulation in physiological conditions and in SIAD. In physiological conditions, apelin and AVP are released in balanced proportions from the magnocellular AVP neurons at levels appropriate for plasma osmolality. In the collecting duct of the kidney, AVP acts on V2-R to increase cAMP production and aquaporin-2 (AQP-2) insertion, leading to water reabsorption. Conversely, apelin, through its action on apelin-R, has the opposite effect. In physiological conditions, water reabsorption is adequate and plasma sodium concentrations are normal. In SIAD, AVP release is excessive relative to plasma osmolality, leading to excessive water reabsorption by the kidney and hyponatremia. Tolvaptan blocks the effect of AVP on V2-R and corrects hyponatremia in SIAD. We hypothesize that the activation of apelin-R with a metabolically stable K17F analog, LIT01-196, would also correct water homeostasis in this disease. This figure was modified with authorization from Llorens-Cortes and Moos^54^, available at https://www.neuroendo.org.uk/doc/briefing_43.pdf.

Arginine-vasopressin (AVP or antidiuretic hormone, ADH) type 2 receptors (V2-R) are expressed in the kidney collecting ducts (CDs). By acting on these receptors, AVP increases cAMP production, leading to the insertion of aquaporin-2 (AQP-2) into the apical membrane of CD, allowing water reabsorption and decreasing urine output (Fig. 1)^6^. In SIAD, plasma AVP levels are inappropriately increased with respect to plasma osmolality^1^. V2-R antagonists, such as tolvaptan, block the stimulation of V2-R by AVP, and have been shown to correct hyponatremia efficiently in rodents^7^ as well as in humans with SIAD^8–10^. Expected effects of aquaretic agents such as intense thirst, polyuria (24 h diuresis up to 6 L per day) and nocturia were observed^11^. Tolvaptan at the dose of 7.5 mg per day was also reported to carry a significant risk of overly rapid sodium correction in 23% of hyponatremia patients having baseline serum sodium concentration <125 mmol/l^12^. These patients exhibit the highest risk of developing osmotic demyelination syndrome when overly rapid correction of hyponatremia occurs^13,14^. Chronic treatment with V2-R antagonists has also shown efficacy in autosomal dominant polycystic kidney disease (ADPKD)^15^. However, aquaresis-related events led 8% of patients with ADPKD receiving tolvaptan to discontinue treatment^15–17^. Therefore, the development of therapeutic agents acting on different targets with a different mode of action could be useful for the treatment of disorders linked to excessive AVP secretion such hyponatremia and ADPKD.

Apelin is a neuro-vasoactive peptide identified as the endogenous ligand of the human orphan G protein-coupled receptor, APJ^18,19^. Following the identification of its endogenous ligand, this receptor was renamed the apelin receptor (apelin-R). Three molecular forms of apelin have been identified in vivo as follows: apelin-36, apelin-17 (K17F), and the pyroglutamyl form of apelin-13 (pE13F)^20,21^. K17F has an affinity 16 times higher than that of pE13F for apelin-R (*K*_i_, 0.05 versus 0.8 nmol/l), but both peptides inhibit similarly forskolin-induced cAMP production in cells expressing the apelin-R. K17F is, however, 20 times more efficient than pE13F for inducing rat apelin-R internalization and β-arrestin mobilization^22,23^. Apelin and apelin-R^24,25^ are strongly expressed in the supraoptic and paraventricular hypothalamic nuclei, in which double-labeling studies have shown them to colocalize with AVP in magnocellular vasopressinergic neurons^20,24–26^. The central administration of K17F in lactating rats, which display an increased phasic electrical activity of magnocellular vasopressinergic neurons and an increased AVP release from the posterior pituitary into the bloodstream, inhibits the activity of these neurons, leading to a decrease in circulating AVP levels and an increase in aqueous diuresis^20^. Apelin-R is also expressed in the kidney, both in the glomeruli and in all nephron segments, including CD-expressing AVP V2-Rs^27^. The intravenous (i.v.) injection of K17F into lactating rats, by decreasing AVP-induced cAMP production, inhibits the AVP-induced AQP-2 insertion into the apical membrane of CD and increases aqueous diuresis^28^. These data were recently strengthened by the observation in a highly differentiated mouse cortical CD cell line (mpkCCD) expressing the V2-R and the apelin-R that apelin-13 decreases the (Deamino-Cys^1^,d-Arg^8^)-vasopressin (dDAVP)-induced phosphorylation and apical membrane expression of AQP-2 after 30–60 min of treatment, and decreases dDAVP-induced AQP-2 mRNA and protein expression after 8–24 h of treatment^29^. This suggests that the aquaretic effect of apelin is not only due to a central effect involving an inhibition of AVP release into the bloodstream, but also to a direct effect in the CD of the kidney, counteracting the antidiuretic effect of AVP mediated by V2-Rs (Fig. 1)^30,31^. Furthermore, plasma AVP and apelin levels are conversely regulated by osmotic stimuli as well in rodents as in humans to maintain body fluid homeostasis^20,21,32^. In hyponatremia patients with SIAD^33^, plasma copeptin levels (a biomarker of AVP release into the bloodstream in humans) are high and inappropriate for plasma sodium levels^33^. In addition, sex- and age-adjusted, plasma apelin concentrations were 26% higher in SIAD patients than in healthy subjects. Consequently, the balance between plasma apelin and AVP levels is not reached, as shown by the apelin to copeptin ratio as a function of natremia that was outside the 95% predicted physiological limits for plasma sodium in 86% of SIAD patients^33^. This imbalance contributes to the corresponding water metabolism defect in hyponatremia patients with SIAD (Fig. 1).

**Fig. 2:**
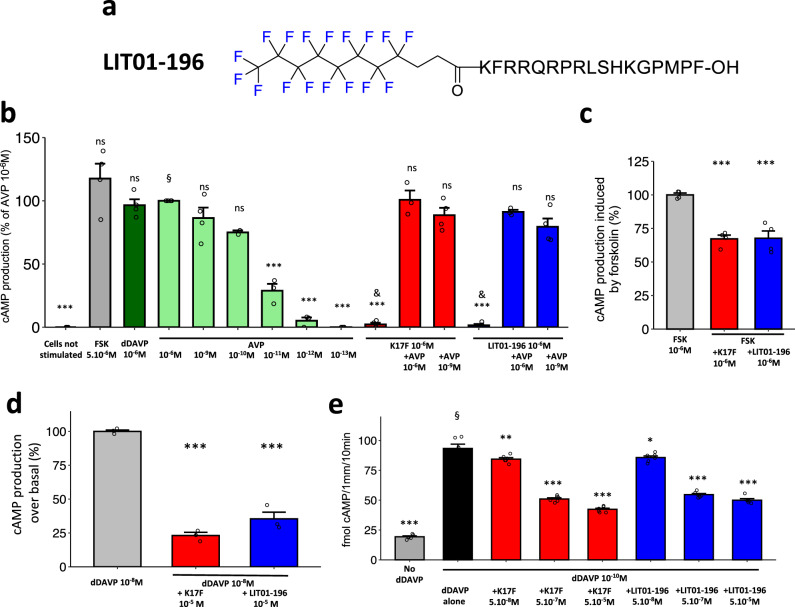
In vitro and ex vivo effects of LIT01-196 on forskolin- or dDAVP-induced cAMP production. **a** Chemical structure of LIT01-196. **b** Effects of forskolin (FSK, gray bar, 5 µmol/l), dDAVP (dark green bar, 1 µmol/l), AVP (light green bars, different concentrations), K17F (red bars, 1 µmol/l) and LIT01-196 (blue bars, 1 µmol/l), and of the combination of either K17F (1 µmol/l) or LIT01-196 (1 µmol/l) with AVP (1 µmol/l and 1 nmol/l) on cAMP production in HEK-293 T cells expressing the human V2-R. Results are expressed as the percentage of increase from basal levels (cells not stimulated) induced by AVP (1 µmol/l). Four individual determinations were performed, expressed as the mean ± SEM. Each point was compared to AVP (1 µmol/l); & denotes the absence of statistical difference with basal levels (cells not stimulated). **c** Effects of K17F (red bars, 1 µmol/l) and LIT01-196 (blue bars, 1 µmol/l) on forskolin (1 µmol/l)-induced cAMP production in mpkCCD cells for 30 min at 37 °C. Data points represent four independent experiments performed in quadruplate and are expressed as the mean ± SEM. **P < 0.01 when compared to forskolin alone. **d** Effect of K17F and LIT01-196 treatment on dDAVP-induced cAMP production. mpkCCD cells grown on permeable inserts were treated with depletion medium containing 10 nmol/l of dDAVP (gray bar) or in combination of dDAVP and 10 µmol/l K17F (red bar) or LIT01-196 (blue bar) for 60 min at 37 °C. Data points represent three independent experiments performed in quadruplate and are expressed as the mean ± SEM. ***P < 0.001 when compared to dDAVP alone. **e** Effects of 0.1 nmol/l dDAVP on cAMP production in microdissected rat outer medullary collecting ducts in the presence or absence of different concentrations (50 nmol/l, 500 nmol/l, and 50 µmol/l) of K17F (red bars) or LIT01-196 (blue bars). Six individual determinations were performed. Each point was compared to dDAVP (10^−6^ mol/l). § denotes a statistical difference (*p* < 0.001) with basal levels (No dDAVP). Groups were compared using ANOVA with Tukey’s adjustment for multiple comparisons for figures (**b**–**e**). n.s. Not significant.

We therefore hypothesized that the supplementary activation of the apelin-R by a metabolically stable K17F analog could counteract AVP-induced water reabsorption and correct hyponatremia. Because the half-life of K17F is short (in the minute range in vivo), we generated LIT01-196 (Fig. 2a), a metabolically stable K17F analog, by adding a fluorocarbon chain to the N-terminal part of K17F, to increase its plasma half-life (>24 h versus 4.6 min for K17F)^22^. LIT01-196 has a subnanomolar affinity for apelin-R and in vitro pharmacological profile similar to that of K17F^22^.

In this study, we aimed (1) to determine the in vivo half-life of LIT01-196 in the blood circulation and its capacity to enter the brain following systemic administration, (2) to verify the efficiency of LIT01-196, as compared to K17F, to inhibit dDAVP-induced cAMP production in freshly microdissected CD, (3) to define if LIT01-196 decreases the apical cell surface expression of phosphorylated AQP-2 in mpkCCD cells, (4) to determine the metabolic effects of LIT01-196 administered subcutaneously (s.c.) in alert rats, and (5) to assess whether the activation of the apelin-R by LIT01-196 decreases AVP-induced antidiuresis and improves hyponatremia in a rodent hyponatremic model, using tolvaptan as a reference aquaretic agent. Here, we show in a rat experimental model of AVP-induced hyponatremia that the s.c. administration of LIT01-196 for 2 days blocks the AVP-induced decrease in urine output and increase in urinary osmolality, leading to a progressive improvement of hyponatremia. These results provide evidence for a major role of apelin, together with AVP, in the regulation of body fluid homeostasis and suggest that the use of apelin-R agonists represents an original approach for the treatment of hyponatremia.

## Results

### Ability of LIT01-196 to bind and activate apelin-R

We first evaluated the ability of the new batch of LIT01-196 (300 mg), synthesized in large quantities for in vivo experiments, to bind to apelin-R and to inhibit forskolin-induced cAMP production in Chinese hamster ovary (CHO) cells stably expressing rat apelin-R-EGFP. As previously reported^22^, LIT01-196, like K17F, bound to rat apelin-R with an affinity in the subnanomolar range (*K*_i_ values for K17F and LIT01-196 of 0.14 ± 0.02 and 0.34 ± 0.05 nmol/l, respectively) (Fig. S1a). In CHO cells stably expressing apelin-R-EGFP, LIT01-196 inhibited the cAMP production induced by 5 µmol/l forskolin with an half-maximal inhibitory concentration (IC_50_) of 1.7 ± 0.5 nmol/l, whereas the IC_50_ for K17F was 0.6 ± 0.2 nmol/l (Fig. S1b), as previously described^22^.

To ensure the selectivity of action of LIT01-196 on the apelin-R and not on the V2-R, we assessed whether LIT01-196 modified cAMP production induced by V2-R stimulation. In HEK-293 T cells expressing the human V2-R, we observed that both LIT01-196 and K17F at a supramaximal concentration of 1 µmol/l did not modify cAMP production induced by 1 µmol/l AVP. Furthermore, K17F or LIT01-196 alone at 1 µmol/l did not modify basal cAMP production in these cells (Fig. 2b). We also verified that K17F and LIT01-196 were able to inhibit forskolin-induced cAMP production in a highly differentiated murine renal cortical CD principal cell line (mpkCCD cells). We found in these cells that K17F (1 µmol/l) and LIT01-196 (1 µmol/l) significantly inhibit cAMP production induced by forskolin (1 µmol/l) by 33% and 32%, respectively (Fig. 2c). Finally, we checked whether K17F and LIT01-196 were able to inhibit dDAVP-induced cAMP production in mpkCCD cells. We observed that K17F (10 µmol/l) and LIT01-196 (10 µmol/l) inhibit dDAVP-induced cAMP production by 75% and 79%, respectively (Fig. 2d).

### Effects of K17F and LIT01-196 on dDAVP-induced cAMP production in microdissected rat outer medullary collecting ducts

We previously showed that dDAVP, a specific and selective V2-R agonist, induced a maximal increase in cAMP production in rat outer medullary collecting ducts (OMCDs) at a concentration of 0.1 nmol/l, and the half-maximal effective concentration was 35 pmol/l^28^. We show here, in the same experimental conditions, that applying dDAVP (0.1 nmol/l) to microdissected rat OMCDs increased cAMP production (92.6 ± 3.6 versus 19.3 ± 0.8 fmol cAMP/mm tubular length/10 min for dDAVP and baseline cAMP values, respectively, *p* < 0.001, *n* = 6). The co-application of dDAVP (0.1 nmol/l) with K17F or LIT01-196 in increasing concentrations (from 50 nmol/l to 50 µmol/l) progressively and significantly inhibited dDAVP-induced cAMP production by 45% and 41%, respectively, in the OMCDs (Fig. 2e).

### Effects of K17F and LIT01-196 on dDAVP-induced phosphorylation of AQP-2 in mpkCCD cells

**Fig. 3:**
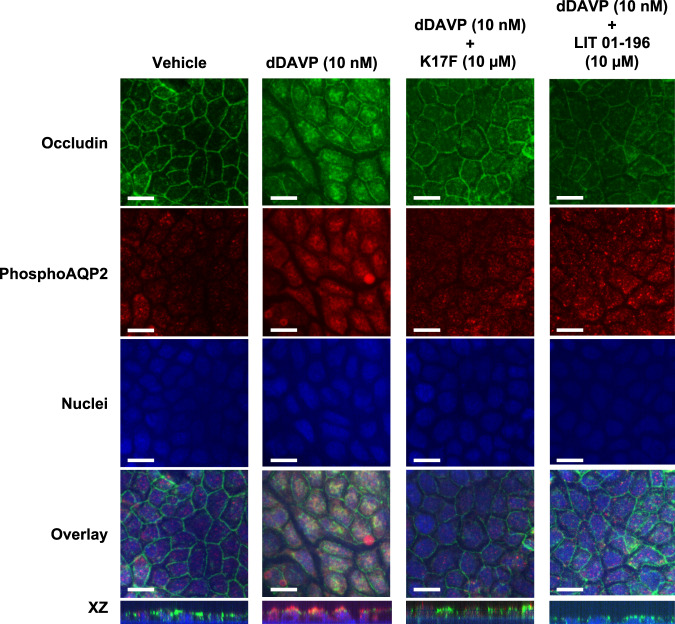
Effect of K17F and LIT01-196 treatment on dDAVP-induced phosphorylation of aquaporine 2. mpkCCD cells were grown on transwell filters for 5 days. After 24 h of starvation, cells were treated with vehicle or 10 nmol/l of dDAVP with or without 10 µmol/l of K17F or LIT01-196. Representative immunofluorescence images of three independent experiments are shown in XY and XZ plans and represent the immunofluorescence staining with an anti-occluding (green) and an anti-phospho-AQP-2 (red) and the staining of the nuclei with DAPI (blue). Bar scale = 20 µm.

As expected from the literature^29,34^, we observed an increase in the phosphorylation of AQP-2 at the apical membrane of the mpkCCD cells treated with 10 nmol/l of dDAVP for 1 h. When mpkCCD cells were co-treated with K17F (10 µmol/l) or LIT01-196 (10 µmol/l), we observed a decrease in the phosphorylation of AQP-2 as shown by the decrease of the immunolabelling of the phospho-AQP-2 (Fig. 3). Intact cell polarity in mpkCCD cells was shown by the tight junction labeling with occludin antibody^35,36^.

### In vivo half-lives of K17F and LIT01-196

**Fig. 4:**
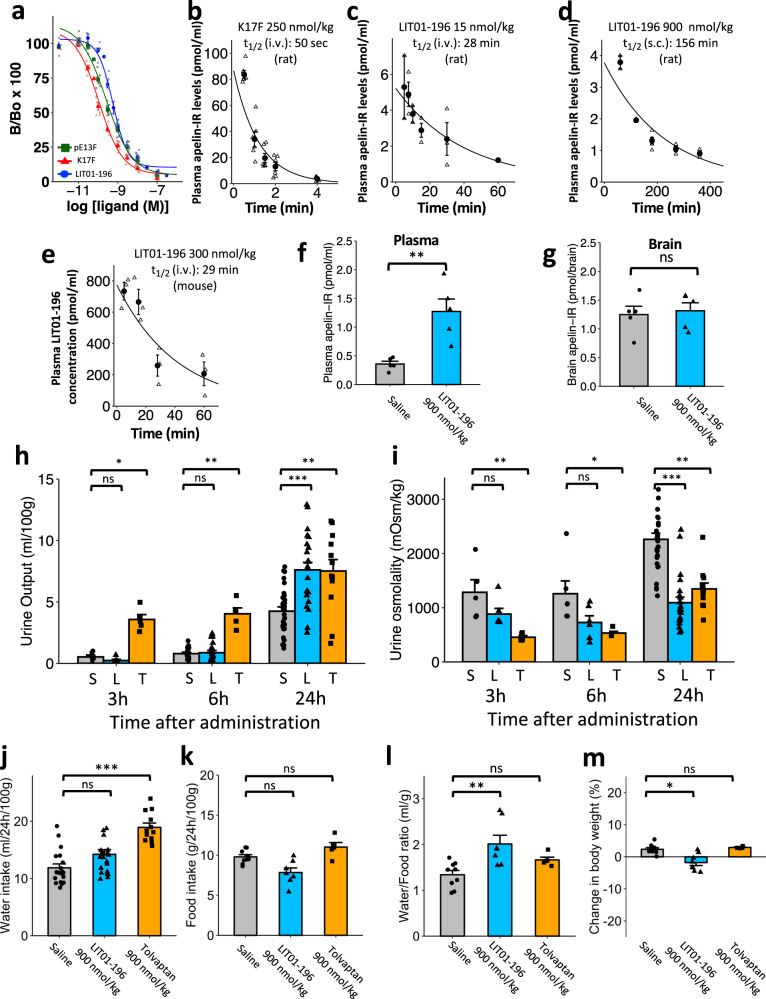
In vivo half-life of K17F and LIT01-196 and effects of s.c. saline, tolvaptan, LIT01-196 on water metabolism, food intake, body weight, and plasma electrolytes in control rats. **a** Inhibition of [^125^I]-pE13F binding to anti-K17F serum (1/4500 final dilution) by unlabeled K17F (six independent experiments, red triangles), pE13F (seven independent experiments, green squares), or LIT01-196 (eight independent experiments, blue circles). Data are expressed as a percentage of the maximal binding of [^125^I]-pE13F in the absence of non-radiolabeled ligand. **b)** In vivo half-life of K17F following i.v. administration in rats. K17F (250 nmol/kg i.v.) was injected in alert rats. **c** In vivo half-life of LIT01-196 following i.v. administration in rats. LIT01-196 (15 nmol/kg i.v.) was administered to alert rats. **d** In vivo half-life of LIT01-196 following s.c. administration (900 nmol/kg) in rats. In figures (**b**)–(**d**), animals (*n* provided in Supplementary Table 1) were killed at various times following injection, trunk blood was collected, and plasma apelin-immunoreactive levels were determined in a radioimmunoassay. **e** In vivo half-life of LIT01-196 following i.v. administration in mice (three animals per time point). LIT01-196 (300 nmol/kg i.v.) was administered to alert mice and plasma LIT01-196 concentration was determined by mass spectrometry analysis. **f** Plasma and **g** brain apelin-immunoreactive levels in rats (*n* = 5 animals per group) 30 min after subcutaneous administration of saline (gray bars, circles) or of LIT01-196 (900 nmol/kg, blue bars, triangles). **h**–**m** In vivo effects of s.c. saline, tolvaptan, and LIT01-196. Evaluation of the effect of saline (1 ml/kg, *n* = 29 animals); LIT01-196 (900 nmol/kg, *n* = 23 animals) and tolvaptan (900 nmol/kg, *n* = 12 animals) in alert male Sprague–Dawley (SD) rats given normal chow on (**h**) urine output and (**i**) urine osmolality measured 3, 6, and 24 h after saline (S, gray bars, circles), LIT01-196 (L, blue bars, triangles), and tolvaptan (T, orange bars, squares). Metabolic parameters were measured 24 h after the s.c. administration of saline, LIT01-196, or tolvaptan (**j**, water intake; **k**, food intake; **l**, water to food intake ratio; **m**, body weight change). Data are shown as mean ± SEM. Multiple comparisons performed by Kruskal–Wallis, followed by post hoc Dunn’s tests with Holm’s adjustment. n.s.: *P* > 0.05, *P < 0.05, **P < 0.01, and ***P < 0.001.

Plasma apelin levels were measured by radioimmunoassay (RIA), with the K17F antiserum that we produced and characterized in a previous study^20^. The antibody directed against K17F recognized K17F, pE13F, and LIT01-196 with a similar affinity (IC_50_: 0.11 ± 0.01 nmol/l for K17F, 0.26 ± 0.03 nmol/l for pE13F, 0.56 ± 0.04 nmol/l for LIT01-196; Fig. 4a). It does not recognize K16P^20^ or Fluoro-K16P (IC_50_ > 1.0 µmol/l for Fluoro-K16P). We determined plasma apelin concentrations at baseline and at different times after the i.v. administration of K17F (250 nmol/kg, i.v.) or LIT01-196 (15 nmol/kg, i.v. or 900 nmol/kg, s.c.) to Sprague–Dawley rats and mice. Plasma apelin concentrations correspond to apelin levels at baseline or to apelin plus LIT01-196-immunoreactive (IR) material after LIT01-196 injection in alert control animals, thus enabling to estimate the in vivo half-life of LIT01-196. We estimated the in vivo half-life of K17F in the rat blood circulation at 50 s (Fig. 4b), accounting for the very brief (2 min) decrease in blood pressure and increase in heart rate observed in rats following the i.v. injection of K17F^37^. In conscious mice, basal plasma apelin levels were 0.98 ± 0.09 pmol/ml. Five seconds after the i.v. injection of K17F (250 nmol/kg), plasma apelin levels had risen to 49.2 ± 7.95 pmol/ml, 50 times basal levels. Plasma apelin concentration decreased rapidly after 60 s, reaching 2.8 times the basal level after 300 s (Fig. S2a). The in vivo half-life of K17F in the mouse blood circulation has been estimated at 44 s, very similar to that obtained in rats (50 s). Both i.v. and s.c. administrations of LIT01-196 to alert rats and mice led to a large increase in plasma apelin-IR levels. We estimated that the in vivo half-life of LIT01-196 in the blood circulation in alert rats was 28 min after i.v. administration (Fig. 4c) and 156 min after a s.c. administration (Fig. 4d). Using mass spectrometry, we evaluated the half-life of LIT01-196 after its injection by i.v. route at the dose of 300 nmol/kg in mice (Fig. 4e), which was estimated at 29 min, close to that obtained in rats after i.v. administration (28 min) using the RIA technique. To explain the higher plasma stability of LIT01-196 as compared to apelin, we explored the possibility that the fluorocarbon chain could bind to plasma proteins, resulting in the protection of the peptide from enzymatic degradation by steric hindrance. By the classical Diamon equilibrium dialysis chamber analysis^38^, we showed that the bound fraction of LIT01-196 to plasma proteins was 69 ± 7% (value reached at equilibrium).

Plasma apelin levels 30 min after s.c. administration of LIT01-196 (900 nmol/kg; Fig. 4f) to Sprague–Dawley rats were 1.3 ± 0.22 pmol/ml, 3.6 times higher than basal levels (0.36 ± 0.05 pmol/ml, *p* = 0.008). Conversely, no change was observed in apelin-IR levels measured in the brain of rats treated with LIT01-196 (1.3 ± 0.14 pmol/brain) compared to basal values obtained in rats treated with saline (1.2 ± 0.15 pmol/brain, *p* = 0.84; Fig. 4g). The increase in plasma apelin-IR was also observed in mice 30 min after s.c. administration of LIT01-196 (900 nmol/kg; Fig. S2b). Plasma apelin-IR levels had risen to 12.4 ± 2.5 pmol/ml, 9.7 times basal levels (1.3 ± 0.073 pmol/ml, *p* = 0.01). At the opposite, no change was observed in apelin-IR levels measured in the brain of mice treated with LIT01-196 (0.440 ± 0.073 pmol/brain) compared to basal values obtained in mice treated with saline (0.391 ± 0.045 pmol/brain, *p* = 0.8; Fig. S2c).

### Effects of K17F and LIT01-196 given by i.v. route on urine output in normonatremic rats

We evaluated the effects on urine output of an i.v. administration of K17F and LIT01-196 in control Sprague–Dawley rats. The i.v. injection of K17F (400 nmol/kg, 855 µg/kg) induced no significant change in 24-h urine output or in urine osmolality in control rats. By contrast, the i.v. administration of LIT01-196 at a dose 27 times lower (15 nmol/kg, 51 µg/kg) induced a 95% increase in urine output (*p* < 0.05) (Fig. S2d) and a 38% decrease in urine osmolality (*p* < 0.05) relative to the values obtained for rats receiving saline i.v. (Fig. S2e). The i.v. administration of K17F or of LIT01-196 did not induce a significant change in water intake (Fig. S2f).

### Effects of LIT01-196 and tolvaptan given by s.c. route on metabolic parameters in normonatremic rats

We first determined in normonatremic rats the optimal dose of LIT01-196 to induce aqueous diuresis. Subcutaneous LIT01-196 from 300 to 900 nmol/kg induced a progressive increase in diuresis and a decrease in urine osmolality. The maximal effect was observed for doses comprised between 900 and 2400 nmol/kg. The effects of LIT01-196 and tolvaptan on urine output and osmolality were similar at an equimolar dose of 900 nmol/kg. However, increasing the dose of tolvaptan to 6700 nmol/kg was associated with an increased effect on urine output and urine osmolality, while increasing the dose of LIT01-196 to 2400 nmol/kg did not significantly increase its aquaretic effect (Fig. S3a, b). We thus used the dose of 900 nmol/kg to study the effects LIT01-196, and compared them to both the equimolar dose of tolvaptan and to a higher dose.

In addition, we compared the kinetics of the effects of LIT01-196 and tolvaptan on metabolic parameters in alert normonatremic Sprague–Dawley rats. Relative to saline (*n* = 29), LIT01-196 (900 nmol/kg, *n* = 23), and tolvaptan (900 nmol/kg, *n* = 12) significantly increased 24-h urine output by 79% and 77%, respectively. Urine osmolality decreased similarly in the two groups, by 52% in animals receiving LIT01-196 and 40% in animals receiving tolvaptan. Although the aquaretic effect of tolvaptan was significant at 3, 6, and 24 h after s.c. administration, the aquaretic effect of LIT01-196 was only significant 12 and 24 h after s.c. administration, showing an effect occurring later with this compound (Fig. 4h, i and Fig. S3c, d). Timing of the aquaretic effect of LIT01-196 depends on its mode of administration since its i.v. administration was previously shown to increase urine output in anesthetized female rats, 2 h after administration^22^.

In order to assess the possibility of diurnal fluctuations of the aquaretic effect of LIT01-196, we compared the effects on urine output and urine osmolality of the s.c. administration of LIT01-196 (900 nmol/kg) administered at 8 a.m. and at 8 p.m. We found that the aquaretic effect of LIT01-196 was independent of the time of administration (Fig. S3c, d).

Increasing the dose of LIT01-196 to 2400  mol/kg did not result in an earlier effect on aqueous diuresis (Fig. S3e, f). The increase in urine output was associated with a significant increase in water intake (Fig. 4j) in the tolvaptan group (+37%) and a slight increase in the LIT01-196 group (+11%). Food (normal chow) intake (Fig. 4k) was similar in the saline and the tolvaptan groups, but slightly and non-significantly lower in animals receiving LIT01-196 (20% decrease). Water intake to food intake ratio was significantly increased by 50% in the LIT01-196 group and non-significantly increased by 24% in the tolvaptan group (Fig. 4l). A slight weight loss (−1.8%) (Fig. 4m) was observed in the LIT01-196 group.

Tolvaptan at a higher dose (6700 nmol/kg, *n* = 13) induced a larger aquaretic effect than LIT01-196 at both 900 and 2400 nmol/kg (Fig. S4a, b), which occurred more rapidly than with LIT01-196 (Fig. S3e, f). LIT01-196 (900 nmol/kg) significantly increased free water excretion fraction by 86%, while tolvaptan (6700 nmol/kg) induced a similar but non-significant increase (Fig. S4c). Sodium excretion fraction (Fig. S4d) was not changed by the administration of LIT01-196 (900 nmol/kg) or tolvaptan (6700 nmol/kg).

### Effects of LIT01-196 and tolvaptan on plasma AVP and apelin levels in control rats

Plasma AVP and apelin (apelin-IR material) levels were measured 3 h after the s.c. administration of saline (*n* = 7), tolvaptan (6700 nmol/kg s.c., *n* = 6), and LIT01-196 (900 nmol/kg s.c., *n* = 7) into alert rats (Fig. S4e–g). Rats receiving tolvaptan had significantly higher plasma AVP levels (147% higher; *P* < 0.001) than control animals receiving saline, while only a modest increase in plasma AVP levels was observed in rats receiving LIT01-196 (37% higher; *P* = 0.021).

Plasma apelin-IR levels in control rats (receiving saline) and in rats receiving tolvaptan were similar. Plasma apelin-IR levels in rats receiving LIT01-196 were significantly higher (185%) than those in control rats (*p* < 0.001). Plasma apelin/AVP ratio was similar in rats receiving tolvaptan or saline, but was higher in rats receiving LIT01-196 (105% higher than in control rats; *p* = 0.038).

### Effects of LIT01-196 on blood pressure, cardiac contractility, kidney function, and blood glucose

Following s.c. injection of LIT01-196 (900 nmol/kg) in alert Sprague–Dawley rats, mean arterial blood pressure (MABP) decreased slightly 3 h after administration, and this change did not reach statistical significance (baseline MABP, 106 ± 2 mmHg, MABP 3 h after LIT01-196 administration, 94 ± 2 mmHg, *p* = 0.063, *n* = 5). MABP measured 24 h after administration of LIT01-196 did not significantly differ from baseline (*p* = 0.88) (Fig. S5a). We also evaluated the effects of s.c. LIT01-196 administration on left ventricular ejection fraction (LVEF) estimated by echocardiography in mice (*n* = 5) and did not observe any significant effect at the slightly higher dose of 1200 nmol/kg (LVEF 3 h after administration, 57.1 ± 0.7 versus 52.6 ± 3.0% before administration, *p* = 0.31) (Fig. S5b). Following the s.c. administration of LIT01-196 at the dose of 900 nmol/kg for 4 consecutive days, we performed histological analysis of the kidneys that did not reveal any abnormality (Fig. S5c–f). Blood creatinine, urea nitrogen, sodium, and potassium levels (Fig. S5g–j) were not changed by LIT01-196 treatment. Repeated administration of LIT01-196 (900 nmol/kg, s.c.) to normonatremic Sprague–Dawley rats was not associated with a change in plasma or urine glucose levels (Fig. S5k, l).

### Establishment of a rodent model of AVP-induced hyponatremia

We established a rodent model of AVP-induced hyponatremia by administering AVP subcutaneously and continuously via an osmotic minipump in male Sprague–Dawley rats placed in metabolic cages and fed a liquid diet. The effects of different doses of AVP on urine output, urine osmolality and plasma sodium, water, and food intake were evaluated. For a dose of 30 or 50 ng AVP/h, urine output decreased during the 4 days following insertion of the osmotic minipump (mean decrease of 61% in rats receiving AVP at a dose of 30 ng/h relative to rats receiving saline), urine osmolality increased by 455%, and plasma sodium concentration decreased by 26% (*p* < 0.001 for urine output, urine osmolality, and plasma sodium). Animals treated with AVP at a dose of 30 ng/h or higher also decreased their food and water intake (*p* < 0.001) (Fig. S6). Little or no effect was observed with a lower dose of AVP (20 ng AVP/h). A moderate decrease in body weight was observed during the experiment in both hyponatremic and normonatremic animals, even in the absence of AVP administration, but weight loss was significantly greater in animals receiving 30 ng/h AVP or more (Fig. S6d). The experiment was stopped after 5 days because some of the animals had lost >20% of their body weight, the acceptable limit established with the ethics committee. The minimum effective dose of AVP (30 ng/h) was therefore used to study the effects of aquaretic agents on AVP-induced hyponatremia.

### Determination of the optimal dose of LIT01-196 in hyponatremic rats

We then determined that the administration of both LIT01-196 and tolvaptan, at the dose of 900 nmol/kg, induced a similar increase in diuresis in the 24 h following s.c. administration in rats receiving 30 ng/h AVP and a liquid diet (increase of 11.7 ± 2.3 and 9.5 ± 2.9 ml/24 h/100 g, respectively). In contrast, urine output did not change 24 h after administration of saline. The use of a lower dose of LIT01-196 (180 nmol/kg s.c.) was associated with a smaller increase of urine output (increase of 1.5 ± 2.0 ml/24 h/100 g), while increasing the dose of LIT01-196 to 2400 nmol/kg did not increase the effect of the compound on urine output (increase of 9.2 ± 4.1 ml/24 h/100 g). In contrast, increasing the dose of tolvaptan to 6700 nmol/kg led to an even greater increase in urine output (increase of 27 ± 9.0 ml/24 h/100 g). Given these results, we used the identical dose of 900 nmol/kg to compare the metabolic effects of LIT01-196 and tolvaptan in a model of AVP-induced hyponatremia.

### Evaluation of the effects of LIT01-196 and tolvaptan in a model of AVP-induced hyponatremia

**Fig. 5:**
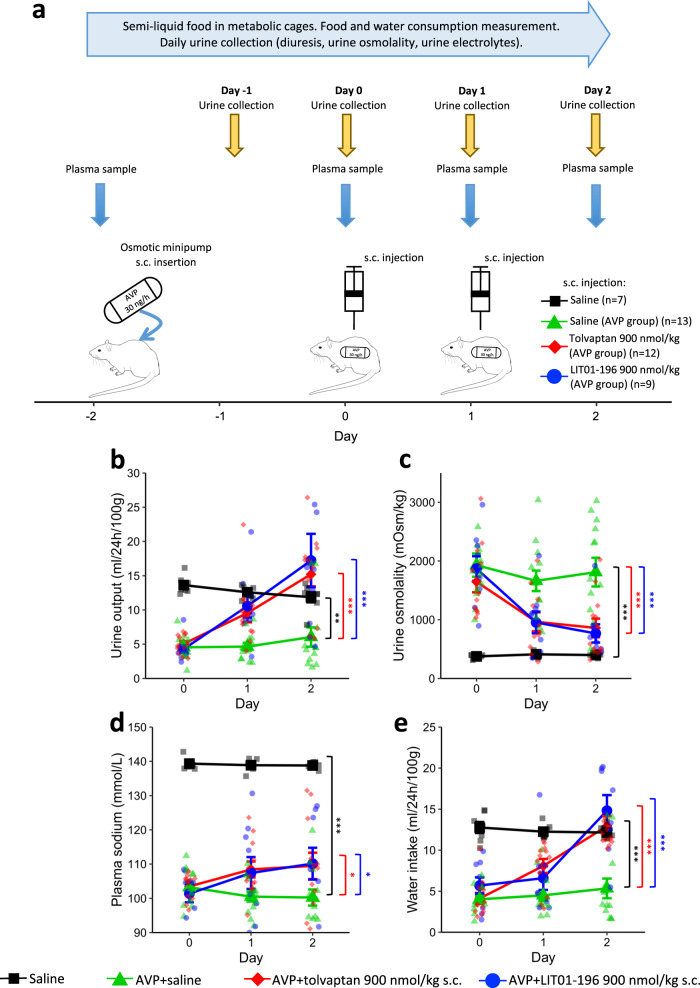
Effects of saline, tolvaptan, and LIT01-196 on water metabolism and plasma electrolytes in hyponatremic rats. **a** Experimental protocol for generating hyponatremia. On day −2, a plasma sample was collected from the tail vein of each animal (male SD rats). Except for the control group, which received no AVP, a subcutaneous osmotic minipump delivering AVP at a rate of 30 ng/h was then implanted into each animal. All the animals were given semi-liquid food. On day 0, a plasma sample was collected and each animal treated with AVP received a s.c. injection of saline (green curve and triangles, 1 ml/kg, *n* = 13 animals), tolvaptan (red curve and diamonds, 900 nmol/kg, *n* = 12 animals), or LIT01-196 (blue curve and circles, 900 nmol/kg, *n* = 9 animals). Animals not treated with AVP received a s.c. injection of saline (black curve and squares, *n* = 7 animals). On day 1, plasma samples were obtained and each animal received another administration of the same treatment. On day 2, a final plasma sample was collected and the animals were killed. Urine output was measured and analyzed every day from days −1 to 2, and water and food intake were also measured daily. Change in 24-h urine output (**b**), urine osmolality (**c**), plasma sodium concentration (**d**), and water intake (including water in the bottle and in the food) (**e**) in animals receiving continuous s.c. AVP at a rate of 30 ng/h from days −2 to 2, and a s.c. injection of saline, tolvaptan, or LIT01-196 on days 0 and 1. Data are shown as mean ± SEM. Each group was compared with the AVP + saline group with a linear mixed-effects model, to take repeated measurements over time into account; *P < 0.05, **P < 0.01, and ***P < 0.001.

As described above, a minipump administering 30 ng AVP/h s.c. was implanted 2 days before the administration of an aquaretic treatment (tolvaptan or LIT01-196) or saline, and all animals received a semi-liquid diet (50% water, 50% gel diet, as described in “Methods”). Water consumption included water mixed with the food as well as water consumed from the bottle (experimental protocol; Fig. 5a). Four experimental groups were studied: the control group (receiving no AVP, *n* = 7), the AVP + saline group (continuous s.c. infusion of 30 ng AVP/h + saline 1 ml/kg s.c. on days 0 and 1, *n* = 13), the AVP + tolvaptan group (30 ng AVP/h s.c. + tolvaptan 900 nmol/kg s.c. on days 0 and 1, *n* = 12), and the AVP + LIT01-196 group (30 ng AVP/h s.c. + LIT01-196 900 nmol/kg s.c. on days 0 and 1, *n* = 9). On day 0, before treatment administration, plasma sodium levels were low and similar in all animals receiving AVP (103 ± 1, 103 ± 1, and 101 ± 3 mmol/l, *p* = 0.6, for the AVP + saline, AVP + tolvaptan, and AVP + LIT01-196 groups, respectively). Animals receiving AVP + saline had significantly lower urine output than controls (49% lower on average between days 1 and 2, *P* = 0.002), higher urine osmolality (355% higher, *P* < 0.001), lower water intake (59% lower, *P* < 0.001), and lower plasma sodium levels (27% lower, *P* < 0.001). These parameters remained stable from days 0 to 2 (Fig. 5b–e).

We then evaluated the effects of LIT01-196 in hyponatremic rats. The model was set up as described above, and LIT01-196 (900 nmol/kg s.c.) was administered on days 0 and 1. Relative to AVP-treated animals receiving saline, the LIT01-196-treated animals had significantly higher urine output (185% higher, on average, from days 1 to 2, relative to the AVP + saline group, *P* < 0.001), and lower urine osmolality (58% lower, *P* < 0.001). An increase in plasma sodium concentration was observed on day 1, 24 h after the first administration of LIT01-196, relative to animals receiving saline (109 ± 5 versus 100 ± 2 mmol/l), and on day 2 (110 ± 5 versus 100 ± 2 mmol/l), and plasma sodium concentration after LIT01-196 administration were significantly higher than in the AVP + saline group (*P* = 0.042). Following treatment with LIT01-196, AVP-treated animals had 177% higher levels of water consumption (including water in diet), on average, from days 1 to 2 (*P* < 0.001, versus the AVP + saline group) (Fig. 5b–e). LIT01-196 treatment was associated with a significant increase in food consumption, and thereby a significant increase in sodium intake. Body weight change from baseline was similar in AVP-treated animals receiving LIT01-196 and saline (Fig. S7). Subcutaneous administration of tolvaptan at the same dose (900 nmol/kg) on days 0 and 1 was associated with a similar effect on urine output, urine osmolality, plasma sodium, and water intake (Fig. 5b–e).

### Plasma AVP and apelin levels following the administration of LIT01-196 or tolvaptan in hyponatremic rats

**Fig. 6:**
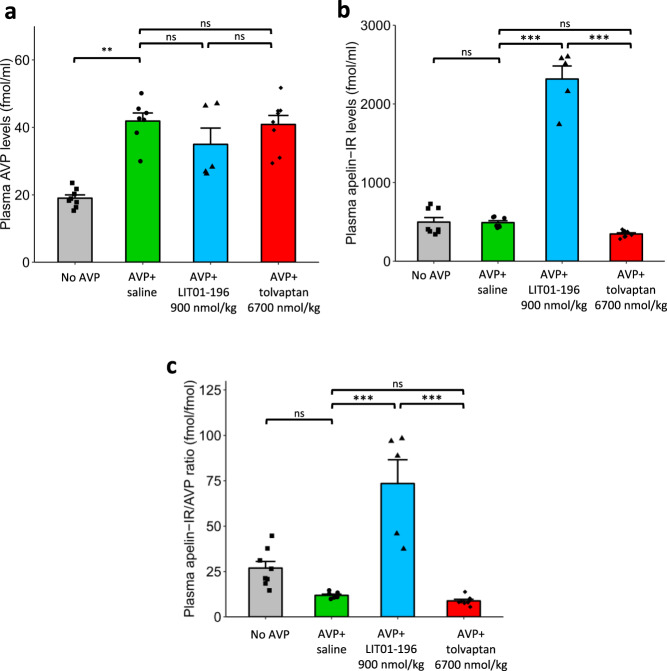
Plasma AVP and apelin levels in hyponatremic rats receiving saline, LIT01-196, and tolvaptan, relative to normonatremic rats. Measurements of plasma AVP levels (**a**), plasma apelin-IR levels (**b**), and plasma apelin-IR/AVP ratios (**c**) in alert male Srague–Dawley rats receiving a semi-liquid diet and no AVP (gray bars, squares, *n* = 8 animals), a 48-h s.c. infusion of AVP at a rate of 30 ng/h together with a s.c. injection of saline (green bars, circles, 1 ml/kg, *n* = 7 animals), LIT01-196 (blue bars, triangles, 900 nmol/kg, *n* = 5 animals) or tolvaptan (red bars, diamonds, 6700 nmol/kg, *n* = 8 animals) 3 h before blood collection. Data are shown as mean ± SEM. Multiple comparisons performed by ANOVA with Tukey’s adjustment for multiple comparisons. n.s.: *P* > 0.05, **P < 0.01, and ***P < 0.001.

Plasma AVP and apelin-IR levels were measured after the continuous administration of AVP (30 ng/h) for 48 h in male Sprague–Dawley rats receiving a semi-liquid diet, 3 h after the administration of saline (AVP + saline), LIT01-196 (900 nmol/kg, AVP + LIT01-196), or tolvaptan (6700 nmol/kg, AVP + tolvaptan) and in rats receiving the same diet but no AVP (Fig. 6). The continuous administration of AVP (30 ng/h) for 48 h significantly increased plasma AVP levels by 120% in animals receiving AVP + saline (Fig. 6a). No significant difference in AVP levels was observed between AVP-treated rats receiving saline or LIT01-196. Plasma apelin levels were similar in animals receiving no AVP and in those receiving AVP + saline, but plasma apelin-IR material levels were 372% higher in rats receiving AVP + LIT01-196 than in rats receiving AVP + saline, because the antibody against K17F recognizes K17F and LIT01-196 with similar affinity (Fig. 6b). In hyponatremic rats, LIT01-196 treatment increased plasma apelin-IR/AVP ratio by 518% relative to rats receiving saline (Fig. 6c). Plasma AVP and apelin levels in rats receiving AVP + tolvaptan were similar to those in animals receiving AVP + saline.

## Discussion

LIT01-196 was previously shown to display, like K17F, full agonist activity for the apelin-R stably expressed in CHO cells, for inhibiting forskolin-induced cAMP production, inducing ERK1/2 phosphorylation, apelin-R internalization, and β-arrestin recruitment^22^. In addition, LIT01-196 has a plasma half-life superior to 24 h versus 4.6 min for K17F^22^. In the present study, we show that LIT01-196, similarly to K17F, dose-dependently inhibits dDAVP-induced cAMP production in CHO cells stably expressing both the apelin-R and the V2-R. In contrast, LIT01-196, even at a supramaximal concentration of 1 µmol/l, does not inhibit AVP-induced cAMP production in HEK-293 T cells, that overexpress V2-R but not apelin-R. This indicates that LIT01-196 does not inhibit dDAVP- or AVP-induced cAMP production by binding to the V2-R and acting as a V2-R antagonist. Hence, LIT01-196 by binding to the apelin-R activates Gi protein coupling, counteracting the increase in cAMP production induced by dDAVP or AVP via Gs protein coupling of the V2-R. Additional evidence that the inhibitory effect of LIT01-196 on dDAVP-induced cAMP production occurs via a non-V2-mediated mechanism is provided by the fact that LIT01-196, like K17F, inhibits forskolin-induced cAMP production in mpkCCD cells, a collecting duct principal cell line expressing both the V2-R and the apelin-R^29,34^. We also show that LIT01-196, similarly to K17F^28^, inhibits the increase in cAMP production induced by dDAVP both in mpkCCD cells and in freshly microdissected rat OMCD, a more physiological preparation. To investigate if the inhibition by LIT01-196 of the cAMP production induced by dDAVP results in a decrease in AQP-2 translocation to the apical membrane, we show in mpkCCD cells that LIT01-196 greatly reduces dDAVP-induced apical cell surface expression of phosphorylated AQP-2. These data are in agreement with previous studies showing that i.v. administration of K17F into lactating rats inhibited the AVP-induced AQP-2 insertion into the apical membrane of the CD and increased aqueous diuresis by decreasing AVP-induced cAMP production in the CD^28^. These data suggested that systemic LIT01-196 administration in rats could induce an increase in aqueous diuresis. Before assessing the in vivo metabolic effects of LIT01-196, we first investigated the in vivo half-life of LIT01-196 in the blood circulation after i.v. administration in alert control mice and rats, by using two methods, mass spectrometry analysis and RIA. Both yielded similar results, 29 and 28 min, respectively, for LIT01-196 versus only 44 and 50 s for K17F after i.v. administration in mice and rats, respectively. We then showed in alert control rats that LIT01-196 had an in vivo half-life in the bloodstream of 156 min after s.c. administration. The increase of the in vivo half-life of LIT01-196 in the blood circulation is probably due to the binding of LIT01-196 to plasma proteins leading to the protection from enzymatic degradation and the reduction of renal clearance, as LIT01-196 has a 69% binding to plasma proteins. This led to evaluate the effects of LIT01-196 on water metabolism.

The s.c. administration of LIT01-196 (900 nmol/kg) in control rats increases urine output, decreases urine osmolality, modestly increases water intake, water intake/food intake ratio, and increases free water clearance while fractional sodium excretion is unchanged, demonstrating an aquaretic effect. This effect of LIT01-196 is independent of the circadian rhythm since similar aqueous diuresis is observed following administration of LIT01-196 at 8 a.m. or 8 p.m. These data are similar to those obtained with tolvaptan, used as a positive control, and administered at an equimolar dose, except for the increase in water intake, which is higher with tolvaptan (+37%) than with LIT01-196 (+11%). The slight increase in water intake induced by LIT01-196 could be related to the fact that intracerebroventricular (i.c.v.) administration of apelin-13 in water-deprived rats reduces water intake^24^, although it increases water intake in normally hydrated mice^39^. Moreover, in patients with primary polydipsia, plasma apelin levels are lower than in healthy volunteers^40^. Altogether, these results suggest that activation of the apelin receptor may limit water intake associated with aquaresis, without abnormally increasing plasma osmolality.

As we previously showed that i.c.v. administration of K17F, by reducing the release of AVP from the posterior pituitary into the bloodstream, increased diuresis in lactating rats^20^, it was important to determine whether LIT01-196 given by s.c. route acted through central and/or renal action. Especially, we wanted to determine if LIT01-196 could cross the blood–brain barrier (BBB) after systemic administration. Neuronal apelinergic fibers have been visualized in the lamina terminalis of rats, along the anteroventral third ventricle region, including the subfornical organ (SFO), the organum vasculosum and the median preoptic nucleus^25^. These structures are all interconnected and project both excitatory and inhibitory signals to the SON and PVN^41^ to regulate the activity of AVP/apelin magnocellular neurons, which also express apelin-R^20,24^. We show here in rats and in mice that 30 min after s.c. injection of LIT01-196 (900 nmol/kg), the compound is abundant in the blood circulation, but absent in the brain, showing that LIT01-196 does not cross the BBB and does not enter the brain. Furthermore, knowing that among the circumventricular organs, only the SFO expresses apelin-R mRNA^42^ and that the administration of LIT01-196 by s.c. route at the dose of 900 nmol/kg does not decrease AVP release in the blood circulation, our results suggest that LIT01-196 given by s.c. route at this dose does not act on the SFO to regulate magnocellular vasopressinergic neuron activity and systemic AVP release. Therefore, the observed effects of LIT01-196 administered s.c. on water metabolism result from the action of the compound at the kidney level.

**Fig. 7:**
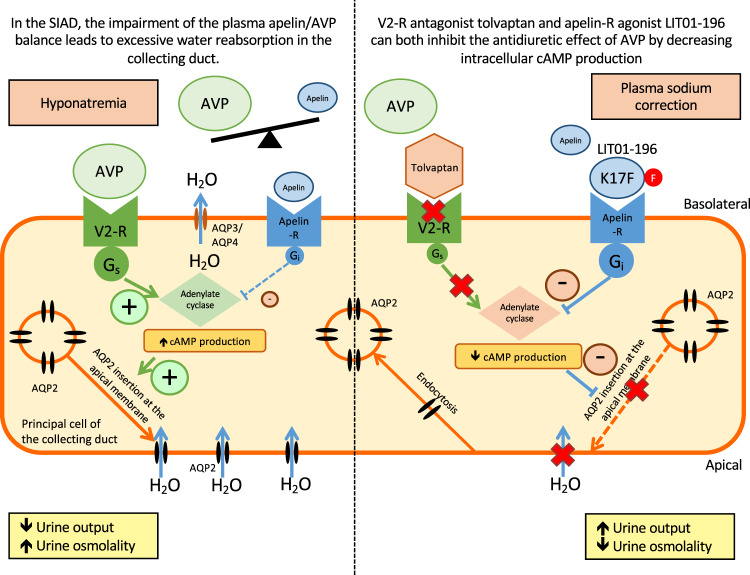
Proposed model of the effects of LIT01-196 on the principal cell of the collecting duct in SIAD. This figure was modified from Flahault et al.^31^, available at https://www.frontiersin.org/articles/10.3389/fendo.2017.00120/full.

As the s.c. administration of LIT01-196 increased aqueous diuresis, we investigated whether LIT01-196 could be used to reverse the antidiuretic effect of AVP in vivo. AVP infusion has been shown to induce hyponatremia efficiently^43,44^, and did not induce excessive mortality^43^. We therefore chose to use AVP to induce hyponatremia in rodents, to obtain an SIAD model resembling the disease observed in humans. We found that a continuous s.c. infusion of AVP (30 ng/h) for four days, together with a semi-liquid diet, led to a decrease in urine output and an increase in urine osmolality. We established that, at this dose, plasma AVP levels were 120% higher than those in rats receiving saline, and that plasma sodium levels were decreased and stable at ~100 mmol/l for 2–4 days after the initiation of AVP infusion. LIT01-196 administered in this rat hyponatremic model at the dose of 900 nmol/kg, for 2 days, via the s.c. route, inhibited the effects of AVP on urine output and urine osmolality efficiently, and induced a progressive correction of plasma sodium levels. Considering the ex vivo and in vivo data, this suggests that LIT01-196, by acting on apelin-R present in the CD, inhibits the AVP-induced cAMP production, thereby inhibiting the insertion of AQP-2 at the apical membrane of the CD, resulting in the inhibition of water reabsorption by the kidney and an increase in aqueous diuresis (Fig. 7).

As expected from the previous studies^7^, tolvaptan also inhibited the antidiuretic effects of AVP. However, while tolvaptan and LIT01-196 at the same dose of 900 nmol/kg resulted in similar aquaretic effects in both control and hyponatremic rats, increasing the dose of tolvaptan resulted in an even larger increase in urine output, while increasing the dose of LIT01-196 did not. Therefore, we cannot exclude a lower clinical efficacy of LIT01-196, as compared to tolvaptan, in the treatment of water metabolism disorders. However, activating the apelin-R and thus modifying the apelin/AVP balance in the collecting duct, rather than blocking the effects of AVP, results in less severe polyuria and may be better tolerated than V2-R antagonists.

The apelin-R is widely expressed, as opposed to the V2-R, which could result in unwanted effects following LIT01-196 administration. Apelin has been reported in rodents to induce a transient decrease in blood pressure^45^, to increase cardiac contractility^46^ and to improve glucose tolerance^47^. However, LIT01-196 (900 nmol/kg) given by s.c. route in Sprague–Dawley rats did not significantly modify MABP, blood glucose levels, and kidney function. LIT01-196 (1200 nmol/kg s.c.) did not modify LVEF in mice. Repeated administrations of LIT01-196 were not associated with renal failure or histological alterations of the kidney, and plasma sodium, potassium, and glucose levels were not affected by LIT01-196 administration in control rats. The various physiological effects associated with the activation of the apelin-R result from the activation of different signaling pathways. Hence, the development of biased apelin-R agonists that specifically target the protein Gi signaling pathway may improve their specificity of action regarding water metabolism.

In conclusion, LIT01-196 is a potent metabolically stable K17F analog that acts as an aquaretic agent. Our model mimics a clinical situation in which the osmotic regulation of circulating AVP levels is impaired, causing SIAD. In this context, LIT01-196 can inhibit the antidiuretic effect of AVP, by increasing urine output, decreasing urine osmolality, moderately enhancing water intake, and progressively correcting hyponatremia. Furthermore, our study constitutes a proof of concept that through the activation of apelin-R, metabolically stable apelin analogs represent promising candidates for the treatment of SIAD. Whether apelin-R agonists could also be beneficial in pathological situations where AVP secretion is in excess and plasma apelin levels are decreased, such as ADPKD^48^, remain to be determined.

## Methods

### Study design

Sample size was determined before the study. For the hyponatremia study, the detection of a mean difference in plasma sodium concentration of 5 mmol/l (expected standard deviation of 3 mmol/l) between the treatment group and the control group would require the inclusion of at least eight animals in each treatment group. AVP-treated animals with an insufficient antidiuretic response (urinary osmolality <850 mOsm/kg) were excluded. The experimental design is described in Fig. 4a. AVP-treated animals were randomly assigned to treatment groups. Blinding was not feasible for treatment administration and sample collection, but the investigators were not aware of the treatment group during sample analysis. Each in vitro experiment was performed at least three times in triplicate.

### Drugs, antibodies, and reagents

K17F was synthesized by PolyPeptide Laboratories (Strasbourg, France). AVP and dDAVP were obtained from Bachem (Bubendorf, Switzerland). Tolvaptan was obtained from Activate Scientific (Prien, Germany). LIT01-196 (300 mg) was synthesized by the Laboratoire d’Innovation Thérapeutique (CNRS UMR7200, Illkirch, France) as previously described^22^. [^125^I]-pE13F (monoiodinated on Lys^8^ with Bolton–Hunter reagent) and [^125^I]-(Tyr2Arg8)-AVP were purchased from PerkinElmer (Wellesley, MA, USA). A specific AVP-[Arg8] antibody was obtained from Peninsula Laboratories International (San Carlo, CA, USA), and used with a 1/4.5 dilution. Rabbit polyclonal antibodies directed against the apelin fragment K17F were produced in the laboratory, as previously described, and used with a 1/4500 dilution^24^. Sodium heparinate, lithium heparinate, and EDTA were obtained from Sigma-Aldrich (Saint-Quentin Fallavier, France). Isoflurane (Isovet) and meloxicam (Metacam) were obtained from Centravet (Nancy, France). Drugs administered s.c. were diluted in 0.9% NaCl (saline, 1 ml/kg) for injection, except for tolvaptan, which was administered in 100% dimethyl sulfoxide (0.2 ml/kg) due to its poor solubility in water. Drugs were administered in the morning (at ~8 a.m.) in all experiments, unless otherwise specified.

### Animals

Male Swiss mice (25–30 g, 4–6 weeks) and male Sprague–Dawley rats (210–230 g, 6–8 weeks) were obtained from Charles River Laboratories (L’Arbresle, France). While housed in our animal facility, they were given free access to normal chow and water, and they were maintained under 12-h light/dark cycles throughout the study. Temperature was maintained between 18 and 22 °C, and humidity between 40 and 60%. When required for i.v. administration, a right femoral venous catheter was implanted in the rats, by tunneling under the skin, under isoflurane anesthesia (4% for induction, 2% for maintenance). Meloxicam (Metacam) was administered postoperatively (1 mg/kg s.c.) for analgesia. All experiments were carried out in accordance with current international guidelines for the care and use of experimental animals, and the experimental protocols were approved by the national animal ethics committee (CEEA, reference numbers 2016-10#3672, 2017-01 #7844 and 01966.02) and ethics regional committee for animal experimentation in Strasbourg (APAFIS reference number 1341#2015080309399690).

### Cell line sources and culture conditions

CHO-K1 cells were obtained from American Type Culture Collection (Rockville, MD, USA) and were maintained in Ham’s F12 medium supplemented with 10% fetal calf serum, 0.5 mmol/l glutamine, 100 U/ml penicillin, and 100 µg/ml streptomycin. HEK-293 T cells were obtained from Cancer Research UK, London Research Institute and were grown in Dulbecco’s modified Eagle’s medium (DMEM) medium supplemented with 10% fetal calf serum, 0.5 mmol/l glutamine, 100 U/ml penicillin, and 100 µg/ml streptomycin. A highly differentiated murine renal cortical collecting duct principal cell (CCD) line, mpkCCDcl4^34^, which had retained the main characteristics of the parental CCDs from which they were derived was used^49^. This cell line was provided by Dr. Michel-Robert Popoff from the Pasteur Institute who previously performed studies on these cells^50^. mpkCCD cells were grown in 1:1 (vol/vol) Ham’s F12:DMEM medium supplemented with 2% fetal calf serum, 20 mmol/l d-glucose, 20 mmol/l HEPES, 1% penicillin/streptomycin, 5 µg/ml insulin, 50 nmol/l dexamethasone, 60 nmol/l sodium selenate, 1 nmol/l triiodothyronine, 10 ng/ml Epidermal Growth Factor, and 5 µg/ml transferrin.

### Microdissection of rat OMCDs

The left kidney of male rats was prepared for nephron microdissection, as previously described^51^. Pieces of OMCDs were isolated under a stereomicroscope.

### Membrane preparations and radioligand binding experiments

Membranes from CHO cells stably expressing rat apelin-R-EGFP were prepared as previously described^23^. We compared the affinities of K17F and the new batch of LIT01-196 by performing classical binding studies with pE13F (Bolton–Hunter radioiodinated on the lysine residue in position 8) as the radioligand. Briefly, membrane preparations (0.5–1 μg total mass of membrane protein/assay) were incubated for 1 h at 20 °C with 20 nmol/l [^125^I]-pE13F in binding buffer alone (50 mmol/l HEPES, 5 mmol/l MgCl_2_, 1% bovine serum albumin (BSA), pH 7.4) or in the presence of pE13F, K17F, or LIT01-196 at various concentrations (0.01 pmol/l to 100 μmol/l). The reaction was stopped by adding ice-cold binding buffer and filtering through Whatman GF/C filters. The filters were washed and radioactivity was counted with a Wizard 1470 Wallac gamma counter (PerkinElmer, Turku, Finland).

### cAMP assay

cAMP assays are described in the Supplementary Information and methods.

### Measurement of phosphorylated AQP-2 immunoreactivity in mpkCCD cells treated by dDAVP, dDAVP + K17F, or dDAVP + LIT

mpkCCD cells were seeded in 12 mm Transwell filters of 0.4 μm pore size at a density of 160,000 cells/cm^2^ and were grown for 5 days. Twenty-four hours before the experiment, cells were starved with depletion media (1:1 (vol/vol) Ham’s F12:DMEM medium). Then, cells were treated at the basolateral compartment with the depletion media (vehicle) or with 10 nmol/l of dDAVP in the presence or in the absence of 10 µmol/l K17F or LIT01-196 for 1 h at 37 °C. The cell culture medium was removed and cells were fixed with 4% formaldehyde in 1× PBS for 10 min at 4 °C, and permeabilized with 0.5% PBS Triton X-100 for 5 min at 20 °C. Then, inserts were incubated in 5% PBS-BSA for 60 min at 20 °C to block non-specific protein binding. Inserts were subjected to immunostaining with a rabbit anti-AQP-2-pS269 antibody (dilution, 1/200, Phospho Solution, Cliniscience, France) and a mouse monoclonal anti-occludin antibody (dilution, 1/200, Santa Cruz, Cliniscience, France) for 16 h at 4 °C. Inserts were then incubated with goat anti-rabbit Alexa Fluor Plus 647 or goat anti-mouse Alexa Fluor plus 488 secondary antibodies (dilution, 1/500, Invitrogen) for 60 min at 20 °C. Inserts were detached and mounted onto microscopy slides with Pro Long Aquafade containing DAPI (4′,6-diamidino-2-phenylindole) (Invitrogen). Slides were analyzed in the *x*–*y* and *x*–*z* axes under a ×63/1.4 Oil WD: 0.17 mm objective with a Zeiss Axioobserver Z1 inverted spinning-disk microscope equipped with a camera sCMOS Hamamatsu 2048 × 2048/pixel size: 6.45 × 6.45 µm^2^ and the lasers (405 nm; 100 mW, 491 nm; 150 mW and 642 nm; 100 mW). Microscope settings were identical for all the samples and experiments were randomized and blinded.

### Apelin and AVP radioimmunoassays

The radioimmunoassays are described in the Supplementary Information and methods.

### Plasma LIT01-196 measurement by mass spectrometry analysis

The mass spectrometry analysis is described in the Supplementary Information and methods.

### Plasma protein binding of LIT01-196

Plasma protein binding of LIT01-196 is described in the Supplementary Information and methods.

### AVP-induced hyponatremia

Hyponatremia was induced with AVP using a protocol adapted from Verbalis et al.^43^. We used male Sprague–Dawley rats that were implemented with an osmotic minipump (ALZET 2001, 200 µl, flow rate of 1 µl/h for 7 days), obtained from Durect Corporation (Cupertino, CA, USA) and filled with a solution of saline and AVP (at various concentrations), which was implanted subcutaneously under isoflurane anesthesia. Meloxicam was administered postoperatively for analgesia. Animals were then individually housed in metabolic cages (Techniplast), and given a semi-liquid diet consisting of 25 ml Gel-Diet Breeding-10 (SAFE, Augy, France) mixed with 25 ml tap water per day, with water available ad libitum. Animal weight, water, and food consumption were measured daily. Urine was collected daily, urinary electrolytes (Na^+^, K^+^, Cl^−^) were determined with an ISE 3000 (Caretium Medical Instruments, Shenzhen, China) and urinary osmolality was measured with a Cryobasic 1 osmometer (Astori Tecnica, Poncarale, Italy). Blood samples were collected on ice, before implantation of the osmotic minipump, and before drug administration, under isoflurane anesthesia, from the tail vein (4 µl of lithium heparinate (1600 IU/ml) for 400 µl of blood). The blood samples were immediately centrifuged (4 °C, 2600 × g, 20 min), and plasma electrolyte (Na^+^, K^+^, Cl^−^) levels were quantified with an ISE 3000. On the last day of the experiment, blood was also collected on ice by intracardiac puncture. This blood sample was collected into lithium heparinate under isoflurane anesthesia; plasma electrolyte levels were determined as described above and the animals were immediately killed by carbon monoxide inhalation. The experimental protocol is summarized in Fig. 4a.

### Plasma glucose levels and fractional excretion of water and sodium

Fractional excretion of water (in %) was calculated using the following formula: (plasma creatinine/urine creatinine) × 100. Fractional excretion of sodium (in %) was calculated using the following formula: (plasma creatinine × urine sodium/urine creatinine × plasma sodium) × 100. Blood and urine creatinine, urea nitrogen, and glucose levels were determined using an AU5800 Beckman Coulter chemistry analyzer (Beckman Coulter, Villepinte, France).

### Histological analyses

Histological analyses methods are described in the Supplementary Information and methods.

### Blood pressure measurements

Blood pressure measurements are described in the Supplementary Information and methods.

### Echocardiographic measurements

LVEF was determined in mice under isoflurane anesthesia at baseline and 3 h after s.c. administration of the compound, as previously described^52^.

### Statistical analysis

We used Mann–Whitney *U* tests or paired Wilcoxon tests for comparisons between two groups, and analysis of variance or Kruskal–Wallis tests followed by post hoc comparisons for comparisons between multiple groups, when appropriate. We used a linear mixed-effects model to compare repeated values recorded over time, with time, treatment group, and the measured parameter as fixed effects and the unique animal identification number as a random effect. We assessed the effects of treatment by performing an analysis restricted to measurements taken after treatment administration. The normality of the data was assessed visually and with the Shapiro–Wilk normality test. The data represented are means, with error bars to indicate the standard error of the mean. The number of experiments or animals used is indicated in each figure legend. Data were collected using Microsoft Excel for MacOS version 16. Statistical analysis was performed with R software version 3.6.3^53^. A *P* value <0.05 was considered significant. All analyses were two-sided. Exact *P* values for all analyses are provided in Supplementary Table 5.

### Reporting summary

Further information on research design is available in the Nature Research Reporting Summary linked to this article.

## Supplementary information

Supplementary Information

Reporting Summary

## Data Availability

The authors declare that the data supporting the findings of this study are available within the Supplementary information files. Source data are provided with this paper.

## Source data

Source Data
