# Supplementary material for: A metabolically stable apelin-17 analog decreases AVP-induced antidiuresis and improves hyponatremia
Source: Nat Commun. 2021 Jan 12;12:305. doi: 10.1038/s41467-020-20560-y (PMC7804859; doi:10.1038/s41467-020-20560-y)
Supplement: Supplementary file 1 — Supplementary Information [file 41467_2020_20560_MOESM1_ESM.pdf]

A metabolically stable apelin-17 analog decreases AVP-induced antidiuresis and improves hyponatremia

Authors: Adrien Flahault, Pierre-Emmanuel Girault-Sotias, Mathilde Keck, Rodrigo Alvear-Perez, Nadia De Mota, Lucie Estéoulle, Sridévi M Ramanoudjame, Xavier Iturrioz, Dominique Bonnet, Catherine Llorens-Cortes

Supplemental file

Supplemental figures
Supplemental tables
Supplementary methods
Supplementary references

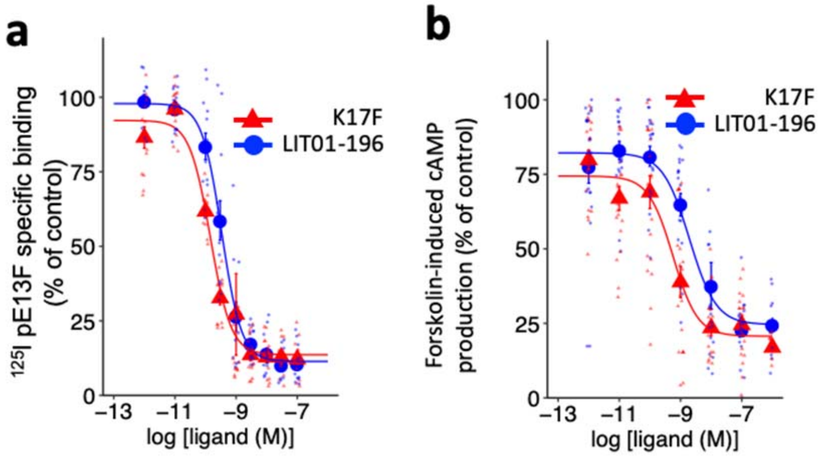

Fig. S1. Pharmacological characterization of K17F and LIT01-196. (a) Competitive binding activities of K17F and LIT01-196 with membranes from CHO cells stably expressing the human apelin receptor. The membranes of CHO cells stably expressing the human apelin receptor (0.5 µg/point) were incubated with 2 x 10^{-10} M [^{125}I]-pE13F in the presence of various concentrations of K17F (red triangles) or LIT01-196 (blue circles). Data are expressed as a percentage of the maximal binding of [^{125}I]-pE13F in the absence of nonradiolabeled ligand and represent the means ± standard error of five independent experiments performed in duplicate. **(b)** Effects of K17F (red triangles) or LIT01-196 (blue circles) on forskolin-induced cAMP production in CHO cells stably expressing rat apelin receptor-EGFP. cAMP production was induced by treating the cells with 10^{-5} M forskolin. The effects of various concentrations of K17F and LIT01-196 on forskolin-induced cAMP production (fmol/10^5 cells) were then evaluated. The data presented are the means ± standard error of six separate experiments.

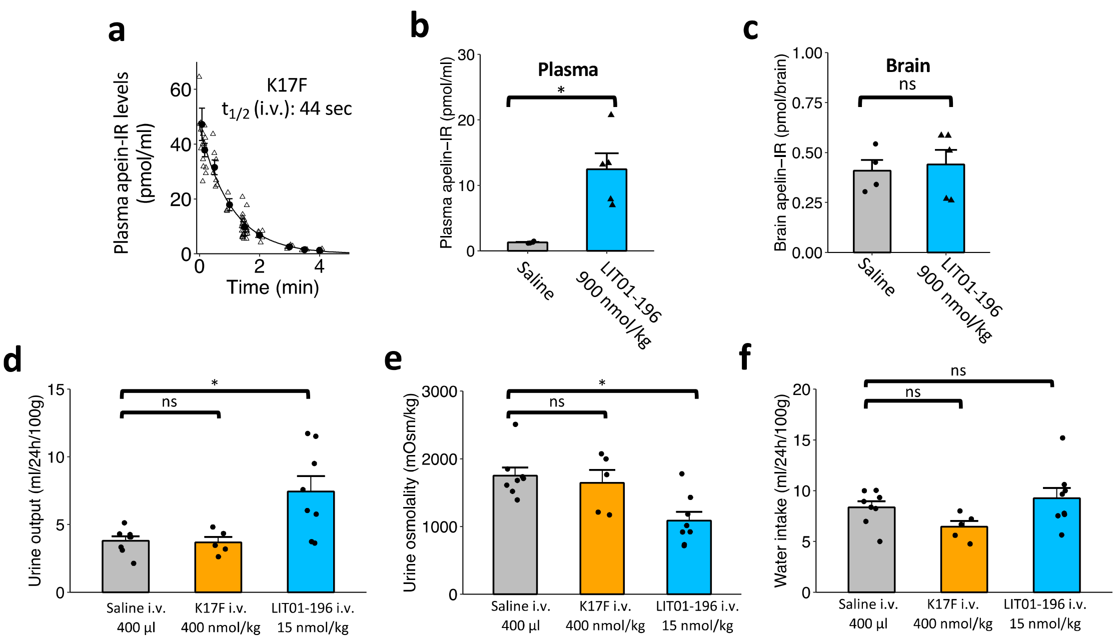

Fig. S2. *In vivo* half-life of K17F in mice, brain penetration of LIT01-196 and its effects on urine output and osmolality in control rats. (a) *In vivo* half-life of K17F following i.v. administration in mice. K17F (250 nmol/kg i.v.) was injected in alert mice. Animals (n is shown in **Supplemental Table 2**) were killed at various times following injection, trunk blood was collected and plasma apelin-immunoreactive levels were determined in a radioimmunoassay. **(b)** Plasma and **(c)** brain apelin-immunoreactive levels in mice (n=5 animals) 30 minutes after subcutaneous administration of saline (grey bars, circles) or LIT01-196 (900 nmol/kg, blue bars, triangles). **(d)** 24-hour urine output, **(e)** urine osmolality and **(f)** water intake measured for 24 hours following the i.v. administration of saline (100 µl/kg, *n*=6 animals, grey bars, circles), K17F (400 nmol/kg, *n*=5 animals, orange bars, circles) or LIT01-196 (15 nmol/kg = 51 µg/kg, *n*=6 animals, blue bars, circles). Data are shown as mean ± S.E.M. Multiple comparisons performed by ANOVA followed by post-hoc Dunnett's tests. Ns: p>0.05, *P<0.05, **P<0.01, ***P<0.001.

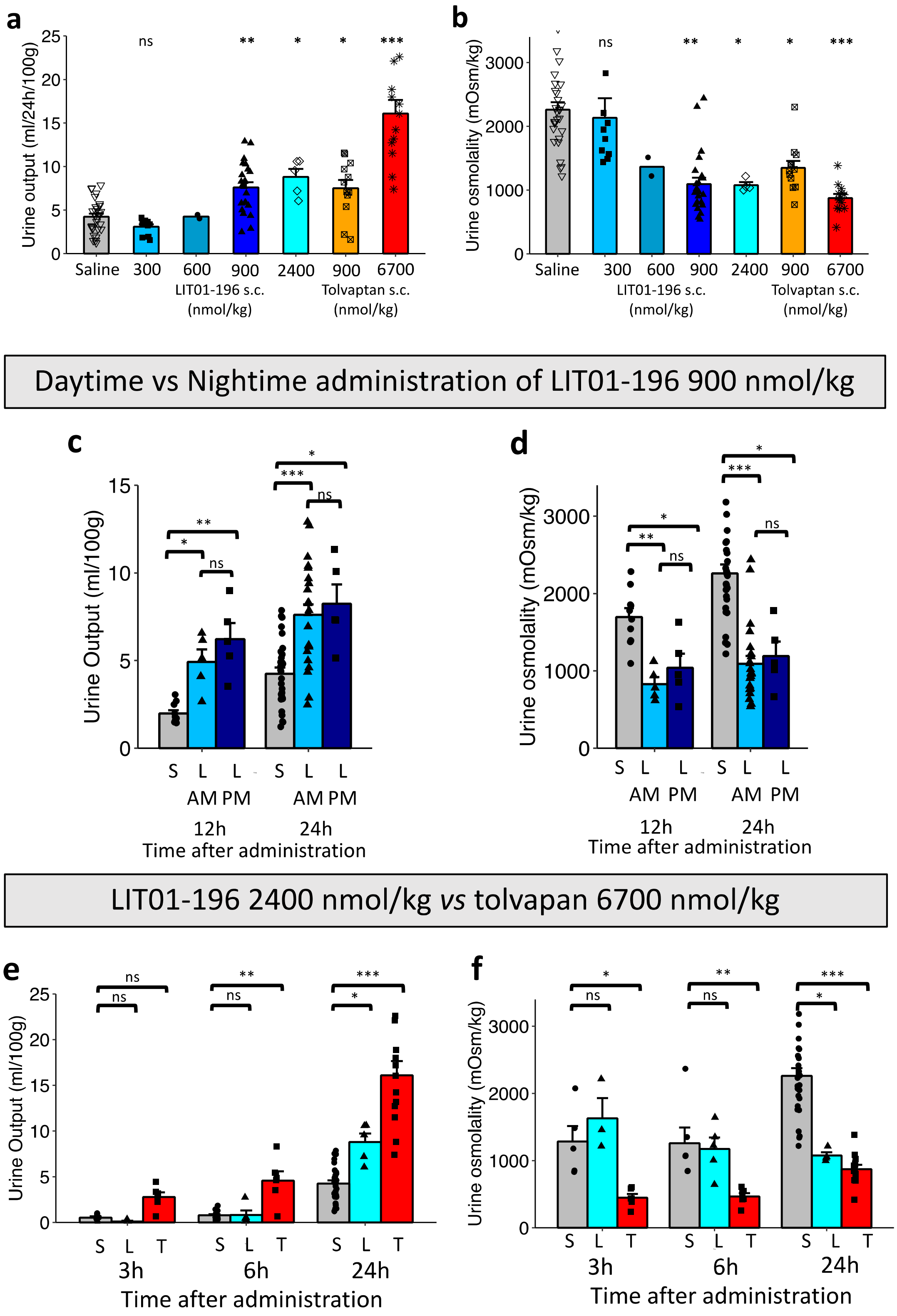

Figure S3. Effects of different doses of LIT01-196 or tolvaptan administered subcutaneously in Sprague-Dawley normonatremic rats. Evaluation of the effect of saline (1 ml/kg, n=19 animals, grey bars, open triangles); various doses of LIT01-196: 300 nmol/kg

(n=11 animals, blue bars, squares), 600 nmol/kg (n=2 animals, cyan bars, circles, no statistical analysis performed), 900 nmol/kg (n=23 animals, dark blue bars, triangles) and 2400 nmol/kg (n=5 animals, light blue bars, open diamonds); and various doses of tolvaptan: 900 nmol/kg (n=12 animals, orange bars, checked squares) and 6700 nmol/kg (n=13 animals, red bars, asterisks) on **(a)** urine output and **(b)** urine osmolality 24 hours after subcutaneous administration. **(c)** Urine output and **(d)** urine osmolality measured 12 and 24 hours after saline (S, grey bars, circles) and LIT01-196 (L) administered at 8 A.M. (blue bars, triangles) or at 8 P.M. (dark blue bars, squares) **(e)** Urine output and **(f)** urine osmolality measured 3, 6 and 24 hours after saline (S, grey bars, circles), LIT01-196 (2400 nmol/kg, L, light blue bars, triangles) and tolvaptan (6700 nmol/kg, T, red bars, squares). Number of animals used for figures C-F are provided in **Supplemental Table 3**. Data are shown as mean ± S.E.M. Multiple comparisons performed by Kruskal-Wallis followed by post-hoc comparisons to the saline group using Dunn's tests with Holm's adjustment. Ns: $p>0.05$, *$P<0.05$, **$P<0.01$, ***$P<0.001$.

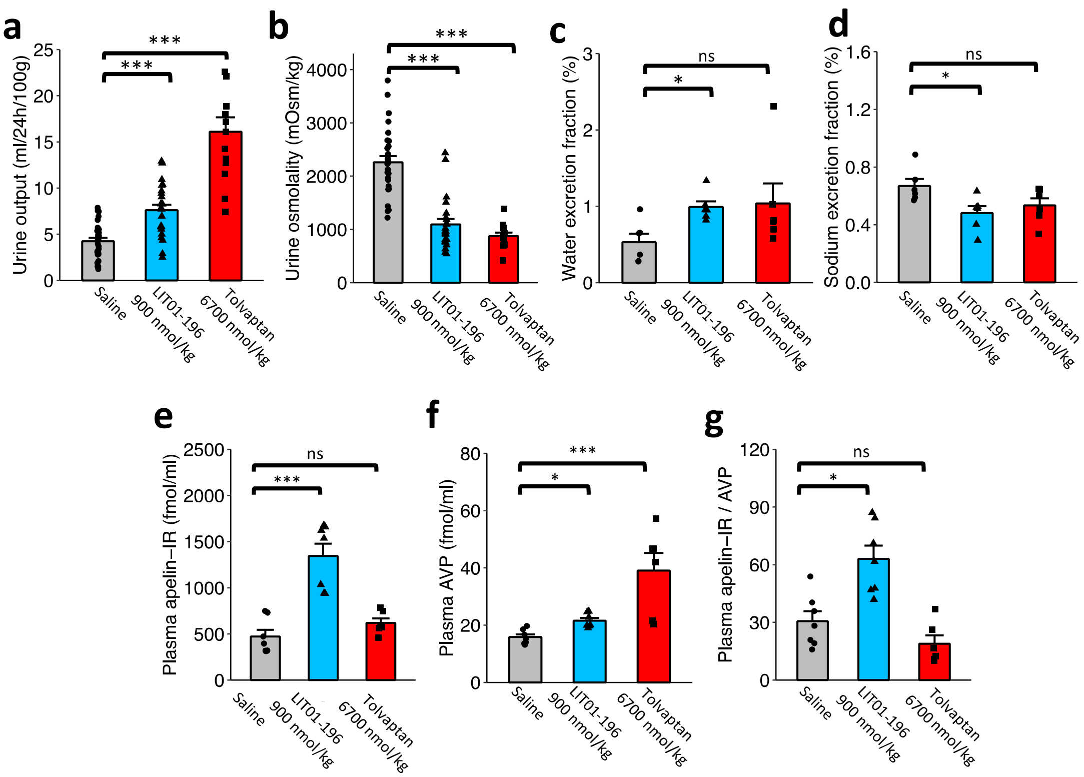

Fig S4. Effects of s.c. saline, LIT01-196 (900 nmol/kg) and a high dose of tolvaptan (6700 nmol/kg) on water metabolism, plasma electrolytes, AVP and apelin-IR plasma levels in control rats. (a) Urine output and **(b)** urine osmolality measured 24 hours after saline (grey bars, circles), LIT01-196 (900 nmol/kg, blue bars, triangles) and tolvaptan (6700 nmol/kg, red bars, squares). **(c)** 24-h water excretion fraction and **(d)** 24-h sodium excretion fraction measured 24 hours after the s.c. administration of saline (1 ml/kg), LIT01-196 (900 nmol /kg) or tolvaptan (6700 nmol/kg) in alert male Sprague-Dawley (SD) rats (number of animals are provided in **Supplemental Table 4**). **(e)** plasma apelin-IR, **(f)** AVP and **(g)** ratio of plasma apelin/AVP levels were determined on plasma samples obtained 3 hours after the s.c. administration of LIT01-196 (*n*=7 animals), tolvaptan (n=6 animals) or saline (*n*=7 animals) in alert male SD rats. Data are shown as mean ± S.E.M. Multiple comparisons performed by Kruskal-Wallis followed by post-hoc Dunn's tests with Holm's adjustment. Ns: $p>0.05$, *$P<0.05$, **$P<0.01$, ***$P<0.001$.

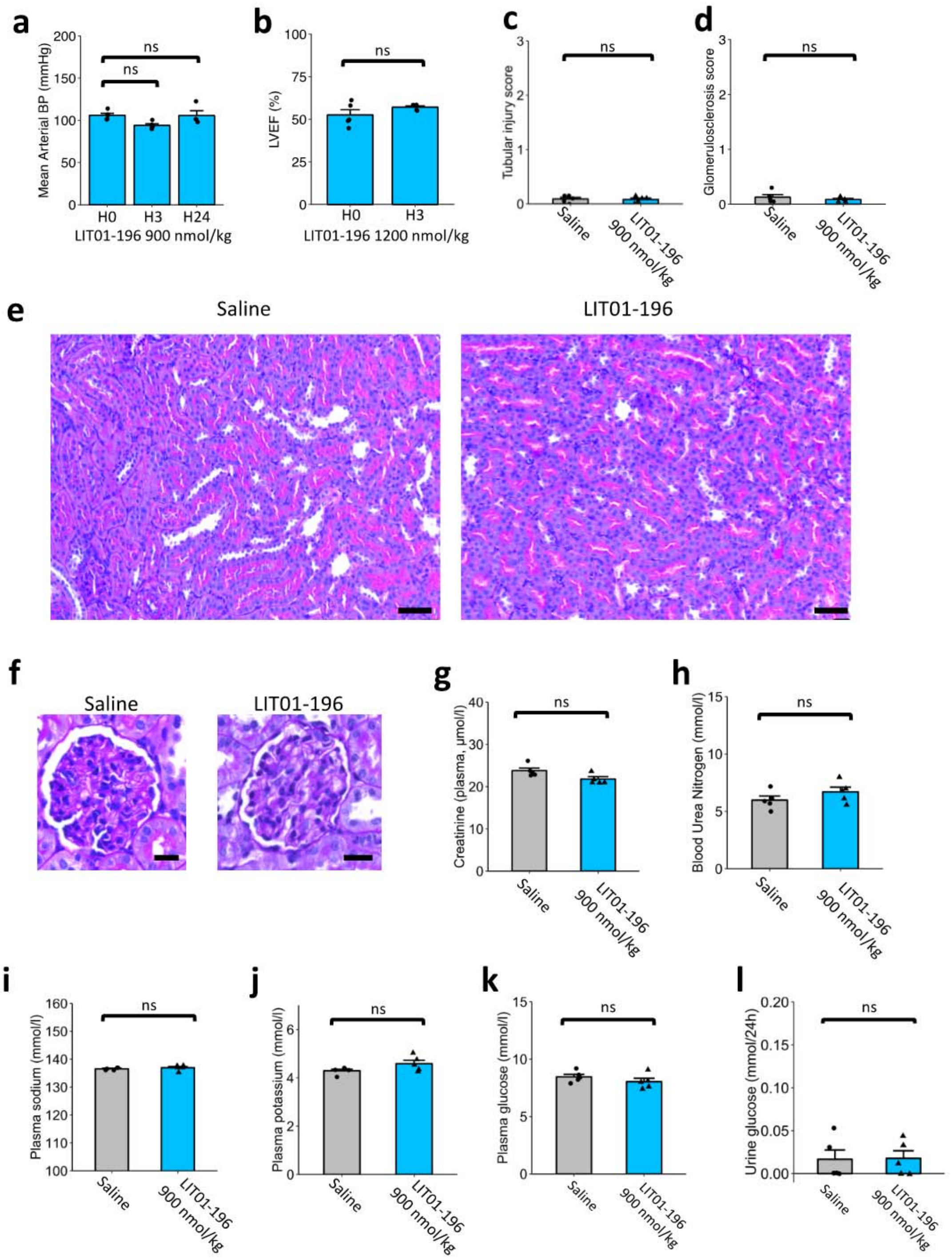

Figure S5. Effects of LIT01-196 on blood pressure, cardiac contractility, kidney function and blood glucose. **(a)** Mean arterial blood pressure (BP) before (H0), 3 (H3) and 24 (H24) hours after s.c. administration of 900 nmol/kg of LIT01-196 in alert normotensive rats (n=5 animals). **(b)** Left ventricular ejection fraction (LVEF) measured by echocardiography in mice (n=5 animals) before (H0) and 3 hours (H3) after s.c. administration of 1,200 nmol/kg of LIT01-196. In figures **c to l**, LIT01-196 (900 nmol/kg s.c., n=5 animals) or saline (1 ml/kg,

n=5 animals) were administered for daily in normonatremic Sprague-Dawley rats during 4 days, and blood and tissues were collected 24h after the last administration. **(c)** Tubular injury score was scored using the percentage of tubules in the outer medulla and corticomedullary junction that showed tubular atrophy or dilatation, loss of brush border, vacuolization, epithelial cell shedding, and denuded tubular basement membrane, as follows: 0, normal; 1, <10%; 2, 10%–25%; 3, 25%–50%; 4, 50%–75%; 5, 75%–100%. **(d)** Glomerulosclerosis score was graded from 0 to 4 according to the percentage of glomerular involvement, with 1 a lesion represented an involvement of 25% of the glomerulus, and a 4 a lesion indicated that 100% of the glomerulus was PAS positive. **(e)** Representative images of periodic acid - Schiff staining of tubular epithelial cells of outer medulla kidney sections from saline and LIT01-196 treated rats. 200x, Bar: 50µm. **(f)** Representative images of periodic acid - Schiff staining of glomeruli from saline and LIT01-196 treated rats. 400x, Bar: 20µm. **(g)** plasma creatinine, **(h)** blood urea nitrogen, **(i)** plasma sodium, **(j)** plasma potassium, **(k)** plasma glucose and **(l)** 24h urine glucose were collected 24 hours after the last administration of saline (grey bars, circles) or LIT01-196 (900 nmol/kg, blue bars, triangles). Data are shown as mean ± S.E.M. Comparisons performed using paired Wilcoxon tests (A-B) or Mann-Whitney U tests (C-D and G to L). Ns: $p>0.05$, *$P<0.05$, **$P<0.01$, ***$P<0.001$.

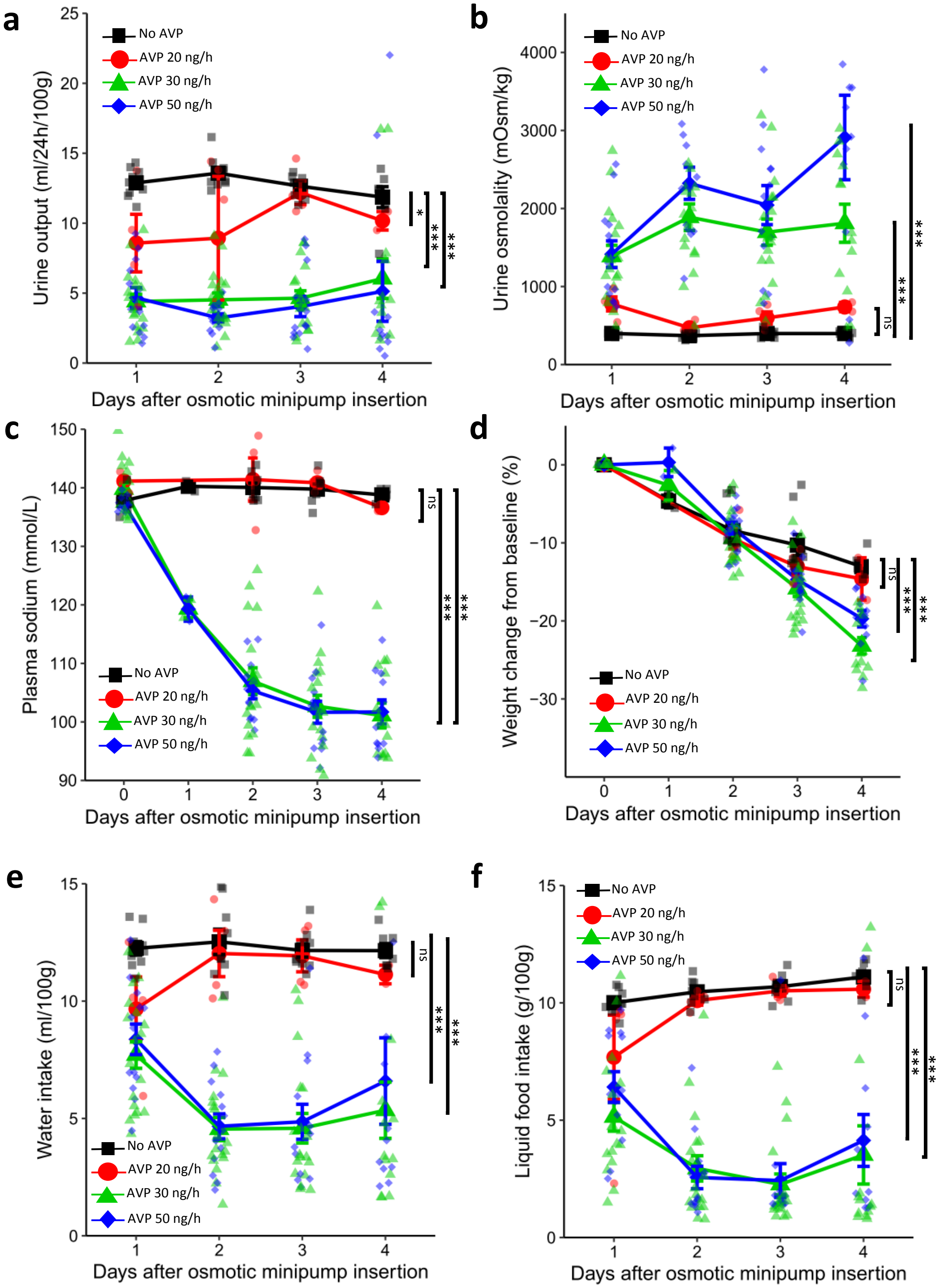

Fig. S6. Determination of optimal AVP infusion rate. Change in 24-hour urine output **(a)**, urine osmolality **(b)**, plasma sodium **(c)**, body weight **(d)**, water intake **(e)** and liquid food intake **(f)** after the insertion of a subcutaneous osmotic minipump delivering AVP at a rate of 20 ng/h (red curves and circles, n=4 animals), 30 ng/h (green curves and triangles, n=17

animals) or 50 ng/h (blue curves and diamonds, n=13 animals) to alert male SD rats, relative to control rats receiving no AVP (black curves and squares, n=9 animals). All animals received a semi-liquid diet. Data are shown as mean ± S.E.M. Each group was compared with the control group from day 1 to day 4, with a linear mixed-effects model, to take repeated measurements over time into account; ns: $P>0.05$, *$P<0.05$, **$P<0.01$, ***$P<0.001$.

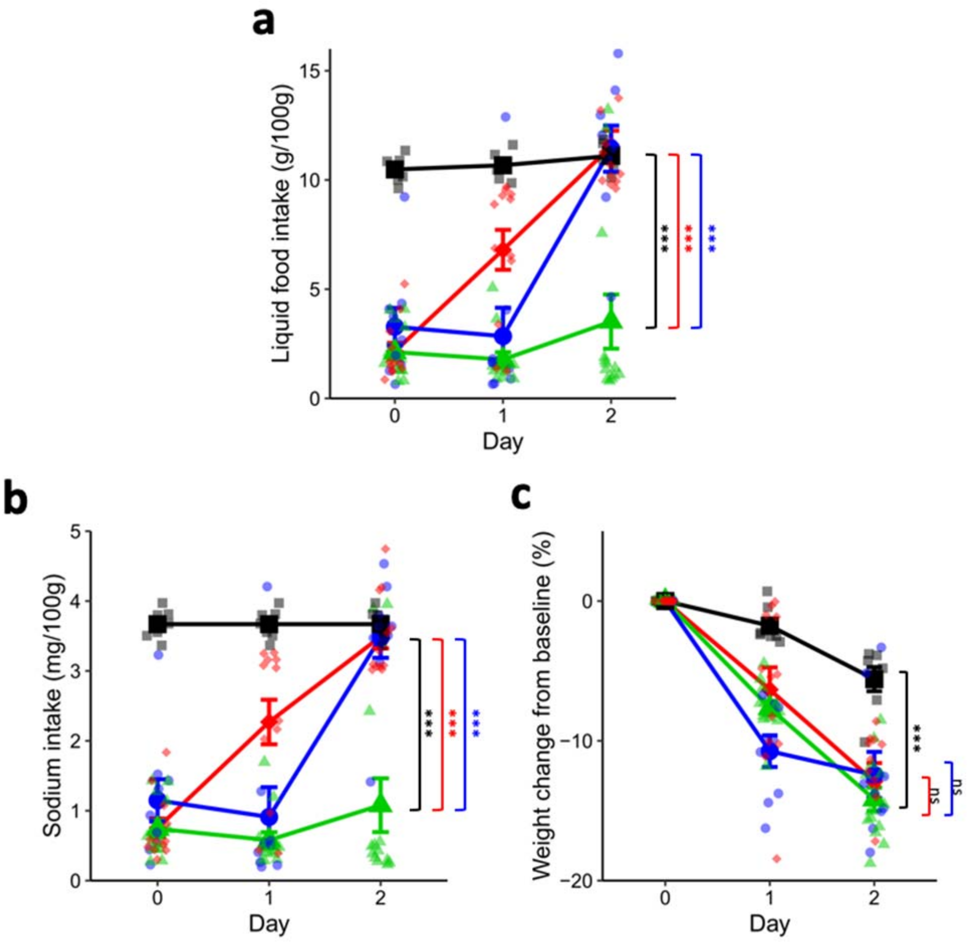

Fig. S7. Effects of saline, tolvaptan and LIT01-196 on water metabolism and plasma electrolytes in hyponatremic rats. (a), change in liquid food intake (without water), **(b)** sodium intake and **(c)** weight change from baseline in animals receiving continuous s.c. AVP at a rate of 30 ng/h from day -2 to 2, and a s.c. injection of saline (green curves and triangles, *n*=13 animals), tolvaptan (red curves and diamonds, 900 nmol/kg, *n*=12 animals) or LIT01-196 (blue curves and circles, 900 nmol/kg, *n*=9 animals) on days 0 and 1. The black curve and squares represents animals receiving no AVP (*n*=7 animals); all animals received a semi-liquid diet. Data are shown as mean ± S.E.M. Each group was compared with the AVP + saline group with a linear mixed-effects model, to take repeated measurements over time into account; ns: *P*>0.05, **P*<0.05, ***P*<0.01, ****P*<0.001.

Supplemental Tables

Figure 4B	Time (min)	0.5	1	1.5	2	4	4
	n (animals)	6	6	6	6	6	6
Figure 4C	Time (min)	5	7.5	10	15	30	60
	n (animals)	2	3	2	3	3	2
Figure 4D	Time (min)	60	120	180	270	360	1440
	n (animals)	2	3	5	3	3	3

Supplemental Table 1. Number of animals per time point for *in vivo* half life determinations in rats (Fig. 4 B-D).

Figure S2A	Time (min)	0.08	0.17	0.5	1	1.5	2	3	3.5	4
	n (animals)	11	6	7	3	30	6	4	3	4

Supplemental Table 2. Number of animals per time point for *in vivo* half life determination in mice (Supplemental Figure 2A).

Group	Saline 12h	LIT01-196 AM – 12h	LIT01-196 PM – 12h	Saline 24h	LIT01-196 AM – 24h	LIT01-196 PM – 24h
Figure S3C	10	5	5	29	23	5
Figure S3D	10	5	5	29	23	5
Group	Saline 3 hours	Saline 6 hours	Saline 24 hours	Tolvaptan 3 and 6 hours	Tolvaptan 24 hours	LIT01-196 All groups
Figure S3E	6	16	29	6	13	5
Figure S3F	6	16	29	6	13	5

Supplemental Table 3. Number of animals per group for the study of metabolic effects of s.c. saline, LIT01-196 (900 nmol/kg) and a high dose of tolvaptan (6,700 nmol/kg) on water metabolism in control rats (Supplemental Figure S3 C-F).

Compound	Saline	LIT01-196 900 nmol/kg	Tolvaptan 6700 nmol/kg
Figure S4A	29	23	13
Figure S4B	29	23	13
Figure S4C	6	6	6
Figure S4D	6	6	6

Supplemental Table 4. Number of animals per group for the study of metabolic effects of s.c. saline, LIT01-196 (900 nmol/kg) and a high dose of tolvaptan (6,700 nmol/kg) on water metabolism, plasma electrolytes, in control rats (Supplemental Figure S4A-D).

Figure	P-values
Figure 2	
2B	Cells not stimulated : <0.001;<br>FSK: 0.56;<br>dDAVP: >0.99;<br>AVP: §:p<0.001; 0.87; 0.32; <0.001; <0.001; <0.001<br>K17F: &:p>0.99/***p<0.001; >0.99; 0.96<br>LIT01-196: &:p>0.99/***p<0.001; >0.99; 0.31
2C	<0.001; <0.001
2D	<0.001; <0.001
2E	No dDAVP <0.001;<br>dDAVP: §:p<0.001;<br>K17F: 0.007; <0.001; <0.001<br>LIT01-196: 0.033; <0.001; <0.001
Figure 4	
4F	0.008
4G	0.84
4H	3h S/L p=0.30; 3h S/T p=0.044<br>6h S/L p=0.68; 6h S/T p= 0.003<br>24h S/L p=0.001 ; 24h S/T p<0.001
4I	3h S/L p=0.20; 3h S/T p=0.002<br>6h S/L p=0.052; 6h S/T p= 0.011<br>24h S/L p<0.001 ; 24h S/T p=0.001
4J	S/L p=0.22; S/T p<0.001
4K	S/L p=0.06; S/T p=0.21
4L	S/L p=0.005 ; S/T p=0.070
4M	S/L p=0.014 ; S/T p=0.32
Figure 5	
5B	S+AVP vs: S p=0.002 ; L p<0.001 ; T p<0.001
5C	S+AVP vs: S p<0.001 ; L p<0.001 ; T p<0.001
5D	S+AVP vs: S p<0.001 ; L p=0.042 ; T p=0.025
5E	S+AVP vs: S p<0.001 ; L p<0.001 ; T p<0.001
Figure 6	

6A	AVP+S vs: No AVP p<0.001; AVP+L p=0.35; AVP+T: p=0.99<br>AVP+L vs AVP+T: p=0.46
6B	AVP+S vs: No AVP p>0.99; AVP+L p<0.001 ; AVP+T: p=0.42<br>AVP+L vs AVP+T: p<0.001
6C	AVP+S vs: No AVP p=0.16 ; AVP+L p<0.001 ; AVP+T: p=0.97<br>AVP+L vs AVP+T: p<0.001
Figure S2	
S2B	0.015
S2C	0.90
S2D	S vs K: p=0.90 ; S vs L: p=0.047
S2E	S vs K: p=0.93 ; S vs L: p=0.030
S2F	S vs K: p=0.11 ; S vs L: p=0.75
Figure S3	
S3A	S vs L300 p=0.54 ; L900 p=0.002 ; L2400 p=0.020 ;<br>S vs T 900 p=0.020 ; T 6700 p<0.001
S3B	S vs L300 p=0.50 ; L900 p<0.001 ; L2400 p=0.001 ;<br>S vs T 900 p=0.006 ; T 6700 p<0.001
S3C	12 hours S vs L-AM p=0.017 ; L-PM p=0.002 ; L AM vs PM p=0.56<br>24 hours S vs L-AM p<0.001 ; L-PM p=0.01 ; L AM vs PM p=0.69
S3D	12 hours S vs L-AM p=0.006 ; L-PM p=0.041 ; L AM vs PM p=0.49<br>24 hours S vs L-AM p<0.001 ; L-PM p=0.012 ; L AM vs PM p=0.71
S3E	3 hours S vs L p=0.19 ; T p=0.068<br>6 hours S vs L p=0.31 ; T p=0.009<br>24 hours S vs L p=0.015 ; T p<0.001
S3F	3 hours S vs L p=0.54 ; T p=0.026<br>6 hours S vs L p=0.95 ; T p=0.008<br>24 hours S vs L p=0.010 ; T p<0.001
Figure S4	
S4A	S vs L p<0.001 ; T p<0.001
S4B	S vs L p<0.001 ; T p<0.001
S4C	S vs L p=0.022 ; T p=0.094
S4D	S vs L p=0.046 ; T p=0.14
S4E	S vs L p<0.001 ; T p=0.26
S4F	S vs L p=0.021 ; T p<0.001

S4G	S vs L p=0.038 ; T p=0.21
Figure S5	
S5A	Baseline vs H3: p=0.0625; H24: p=0.875
S5B	0.31
S5C	0.92
S5D	0.53
S5G	0.068
S5H	0.35
S5I	0.20
S5J	0.22
S5K	0.22
S5L	0.82
Figure S6	
S5A	No AVP vs AVP 20: p=0.024 ; AVP 30: p<0.001 ; AVP 50: p<0.001
S5B	No AVP vs AVP 20: p=0.43 ; AVP 30: p<0.001 ; AVP 50: p<0.001
S5C	No AVP vs AVP 20: p=0.78 ; AVP 30: p<0.001 ; AVP 50: p<0.001
S5D	No AVP vs AVP 20: p=0.20 ; AVP 30: p<0.001 ; AVP 50: p=0.002
S5E	No AVP vs AVP 20: p=0.80 ; AVP 30: p<0.001 ; AVP 50: p<0.001
S5F	No AVP vs AVP 20: p=0.80 ; AVP 30: p<0.001 ; AVP 50: p<0.001
Figure S7	
S7A	S+AVP vs: S p<0.001 ; L p<0.001 ; T p<0.001
S7B	S+AVP vs: S p<0.001 ; L p<0.001 ; T p<0.001
S7C	S+AVP vs: S p<0.001 ; L p=0.64 ; T p=0.33

Supplemental Table 5. Exact P-values for all analyzes, per figure. S: Saline; L:LIT01-196; Tolvaptan.

Supplementary Methods

cAMP assay.

We quantified cAMP production with the cAMP dynamic 2 assay kit (Cisbio Bioassays, Codolet, France), which is based on homogeneous time-resolved fluorescence (HTRF) technology, as previously described [1]. For cells expressing the Apelin-R, 2000 CHO cells/well stably expressing rat apelinR-EGFP (clone B70) were added to the 384-well plate and stimulated with 5 µmol/l forskolin (FSK) and various concentrations (0.01 pmol/l to 100 µmol/l) of K17F or LIT01-196 for 30 minutes at 20°C. For cells expressing the V2-R, HEK-293T cells were transiently transfected with the human V2-R (cDNA Resource Center), after 48h, 4000 cells/well were added to white 384-well optiplates and stimulated either with 5 µmol/l forskolin (positive control) or with dDAVP (1µmol/l) or with various concentrations of AVP (1µmol/l to 0.1pmol/l) or with K17F or LIT01-196 (at 1µmol/l) in absence or in presence of AVP (1µmol/l or 1nmol/l) for 30 minutes at 37°C. Cells were then lysed, and cAMP levels were determined in accordance with the kit manufacturer's instructions. Stimulation was performed in stimulation buffer-IBMX (SBI) (Hank's balanced salt solution (HBSS-Life Technology), 5 mmol/l Hepes, 0.1% BSA stabilizer (Perkin Elmer), 1 mmol/l isobutylmethylxanthine (IBMX, Sigma Aldrich, pH 7.4)). For cAMP assay with mpkCCD cells, the cells were grown on 12 mm Transwell filters (0.4 µm pore size, Corning Costar, Cambridge, MA) until they reach confluency (4-5 days). For forskolin experiments, culture media was replaced with SBI in apical and basolateral compartments. After 30 min of equilibration at 37°C the cells were treated in the basolateral compartment with SBI alone or SBI containing 1 µmol/l forskolin with or without 1 µmol/l K17F or LIT01-196 and incubated for 30 min at 37°C. For the dDAVP experiments, 24h before the experiments the cells were starved with depletion media (1:1 (vol/vol) Ham's F12 : DMEM medium). Then the cells were treated on the basolateral compartments with depletion media containing 1 µmol/l

MLN4760 (an ACE2 inhibitor), 1 µmol/l Thiorphan (a neprilysin inhibitor) and 100 µmol/l bestatin (an aminopeptidase inhibitor) for 30 min at 37°C. Then cells were treated with 10 nmol/l dDAVP with or without 10 µmol/l K17F or LIT01-196 and incubated for 60 min at 37°C. Then the media was removed and 100 µl of lysis buffer (Cisbio Bioassays, Codolet, France) was added to Transwell filter and plate was incubated at 20°C for 2 hours. Cell lysates were diluted 1/500 and 10 µl was subjected to cAMP measurement in white Optiplate 384-well plates (Perkin Elmer) according to manufacturer's instructions (Cisbio Bioassays, Codolet, France). Cyclic AMP levels were measured in microdissected OMCDs by the same method, as previously described [2]. Results are expressed in femtomoles of cAMP produced per mm tubular length and per 10 min incubation time at 37°C.

Apelin radioimmunoassay

Plasma samples (0.5 ml) were acidified with 0.175 ml of 3 mol/l HCl and stored at -80°C until use for apelin RIA. Before thawing the sample on ice, 0.05% BSA was added, and the samples were centrifuged at 20000 x *g* at 4°C for 10 min. The supernatants were collected and the pH was adjusted to 6.5 with 10 mol/l NaOH and 2 mol/l Tris-HCl buffer (pH 7.4). Apelin was extracted from plasma by mixing the supernatant with 0.3 ml of 0.1% BSA in H_2O and 1 ml of 1% trifluoroacetic acid (TFA) - 0.1% BSA, and running the mixture on a Sep-Pak C18 cartridge (Waters) previously washed with 2 ml 100% acetonitrile and equilibrated with 5 ml 1% TFA - 0.1% BSA. The columns then were washed with 3 ml 1% TFA - 0.1% BSA and apelin was eluted with 1.5 ml 100% acetonitrile. The samples were dried and dissolved in 0.32 ml of RIA buffer (19 mM $NaH_2PO_4 \bullet H_2O$, 81 mM $Na_2HPO_4 \bullet 2H_2O$, 50 mM NaCl, 0.1% TritonX-100, 0.01% NaN_3, 0.1% BSA).

For brain apelin RIA, the half brain was homogenized by sonication in 10 volumes of 0.4 N HClO4 supplemented with 0.05% BSA and kept on ice during 1h. The homogenate was centrifuged (17000 x g, 4°C, 20 min). The supernatant (4 ml) was collected, and mixed with

0.4 ml potassium phosphate buffer (0.4 mol/l) and the pH was adjusted to 6.5 with 3 N KOH, and the mixture was left to stand for 15 min on ice and then centrifuged (17000 x g, 4°C, 20 min). Two ml of the supernatant were mixed with 2 ml of 1% TFA - 0.1% BSA and loaded onto a a Sep-Pak C18 cartridge (Waters) previously washed with 2 ml 100% acetonitrile and equilibrated with 5 ml 1% TFA - 0.1% BSA. The columns were then treated as described in the paragraph above.

Plasma or brain apelin levels were determined by RIA. Samples (0.1ml) were mixed with ^{125}I-labeled pE13F (iodinated on Lys8 by the Bolton and Hunter method, 2200 Ci/mmol, Perkin-Elmer, Waltham, MA; 0.05 ml, 19000 dpm) and polyclonal K17F antiserum (0.05 ml, final dilution: 1/4500) to give a total volume of 0.2 ml and were incubated at 4°C overnight. We used this radioligand instead of [Glp65, Nle75, Tyr77] [^{125}I]-apelin13 since the substitution of the C terminal Phe of pE13F was shown to modify the binding affinity of the apelin derivatives as described previously by [3,4].

We then added 0.5 ml of Amerlex (Amersham RPN 510), and the resulting mixture was incubated for 10 min at room temperature. The tubes were centrifuged at 2600 x *g* at 4°C for 20 min. The supernatant was removed and the radioactivity of the precipitates was measured.
We assessed the cross-reactivity of the apelin antiserum with various N- and C-terminally truncated fragments of K17F and several other bioactive peptides. K17F (200% cross-reactivity), pE13F and apelin 36 (100% cross-reactivity) were well recognized by the antiserum, whereas the removal of the phenylalanine residue at the extreme end of the C-terminus of K17F (forming K16P) decreased recognition to barely detectable levels (<0.3% cross-reactivity). Negligible cross-reactivity was observed for angiotensin II, angiotensin III, neuropeptide Y and arginine-vasopressin. This antiserum identified the apelin present in the plasma of rodents as the pyroglutamyl form of apelin 13 (pE13F) and, to a lesser extent, K17F [5] and in the hypothalamus, in major part as pE13F. Most of the apelin in human plasma was

K17F, followed by pE13F, and, to a lesser extent, apelin 36 [6]. The detection limit was 6 fmol/ml, and the quantification limit was 12 fmol/ml. The within- and between-assay coefficients of variation were 3 and 5%, respectively[6]. The recovery (mean±SEM) was 91±3% and the values for apelin levels were corrected for recovery.

We estimated the *in vivo* half-lives of K17F and LIT01-196 with a least-squares fit analysis, according to the following equation: $[\text{plasma apelin}]_{tx} = [\text{plasma apelin}]_{t0} * e^{(-kt)}$, where k is the elimination rate constant.

Plasma LIT01-196 levels determination by mass spectrometry analysis

6 weeks old CD-1 mice were injected with 300 nmol/kg of LIT01-196 via the tail vein (3 animals per time point). Blood samples (400µL) were collected at different time points (5 min, 15 min, 28 min and 60 min) by intracardiac puncture and placed into EDTA-coated tubes, centrifuged 12000 x g at 4 °C for 10 min and the plasma was stored at -80 °C. The samples were analyzed by UHPLC-MS/MS (LC-MS 8030, Shimadzu, electrospray ion source) using a C18 column (Phenomenex 2.6 µm Kinetex, 50 X 2.1 mm) and a linear gradient of solvent B 5-95% v/v over 1.2 min at a flow rate of 0.5 mL/min (solvent A: H2O and 0.05% formic acid; solvent B: acetonitrile). Plasma samples were processed before RP-HPLC analysis as follows: 400 µL of each plasma sample were then mixed with 1 ml of acetonitrile for protein precipitation and compound extraction. Samples were vortex-stirred for 5 min, sonicated for 1 min and centrifuged at 10000 x g for 5 min. 400 µl of supernatants were freeze-dried and then dissolved in 20 µl of water/acetonitrile 1/1. Peptides were quantified by integrating the area under the peaks, and normalization was based on the standard and compared to standard curves. Standard curves were obtained by analyzing known peptide quantities that were dissolved in plasma and processed as well as analyzed using the same procedure. Pharmacokinetics parameters were calculated using a one-compartment model. Peptide injection and blood sample collection were performed by

TechMedILL platform (Illkirch) reviewed and approved by the agriculture ministry regulating animal research in France (Ethics regional committee for animal experimentation Strasbourg, APAFIS 1341#2015080309399690).

AVP radioimmunoassay

Male Sprague-Dawley rats were killed by decapitation, and trunk blood was collected on ice (into 50 µl of 0.3M EDTA (pH 7.4) per 1 ml of blood). Samples were centrifuged (2,600 x g, 4°C, 20 min), and the resulting plasma was split into 500 µl aliquots, to which we added 168 µl of 3N HCl before storage at -80°C. The AVP RIA was performed as previously described [5], using a specific AVP-[Arg8] antibody (final dilution, 1/4.5) and [^{125}I]-(Tyr2Arg8)-AVP (2200 Ci/mmol, Perkin-Elmer, Billerica, MA; 15000 dpm) as a tracer.

Plasma protein binding of LIT01-196

Plasma protein binding of LIT01-196 was determined using Thermo Scientific Single-Use RED Plate (Rapid Equilibrium Dialysis) composed of dialysis inserts with a membrane cutoff of 12 kDa. Compound (10 µmol/l) was spiked in plasma chamber (donor side); phosphate buffer was placed in the receiving side. The plate was rotated at 37 °C on an orbital shaker at 600 rpm for 4 hours. Peptide concentration was determined for the plasma sample that represents the bound fraction, the peptide concentration was determined for the buffer sample that represents the free fraction. The samples were analyzed by UHPLC-MS/MS (LC-MS 8030, Shimadzu, electrospray ion source) using a C18 column (Phenomenex 2.6 µm Kinetex, 50 X 2.1 mm) and a linear gradient of solvent B 5-95% v/v over 1.2 min at a flow rate of 0.5 ml/min (solvent A: H_2O and 0.05% formic acid; solvent B: acetonitrile). Plasma samples (70 µl) were treated with 70 µl of phosphate buffer and 350 µl of acetonitrile to precipitate the proteins. Percentages of bound and free peptides were calculated as follows: % free peptide = (peak area of buffer chamber / peak area of plasma chamber) x 100*;* % bound peptide = 100 - % free.

Histological analyzes

Kidneys were fixed in paraformaldehyde 4% overnight and processed for paraffin embedding. Sections of 4 µm thickness were made and stained with the periodic acid-Schiff (PAS) reagent and counterstained with hematoxylin. Histological analyzes were performed by a trained nephrologist (AF), blinded of the treatment group. Tubular injury score was scored using the percentage of tubules that showed tubular atrophy or dilatation, loss of brush border, vacuolization, epithelial cell shedding, and denuded tubular basement membrane, as follows: 0, normal; 1, <10%; 2, 10%–25%; 3, 25%–50%; 4, 50%–75%; 5, 75%–100%, and was calculated from twenty viewing fields selected randomly from the outer medulla and corticomedullary junction and were examined at ×400 magnification. Glomerulosclerosis score was graded from 0 to 4 according to the percentage of glomerular involvement, with 1 a lesion represented an involvement of 25% of the glomerulus, and a 4 a lesion indicated that 100% of the glomerulus was PAS positive, and was calculated from at least 20 glomeruli selected randomly.

Blood pressure measurements

Rats were implanted with a right femoral artery catheter which was passed beneath the skin under isoflurane anesthesia. Blood pressure (BP) was recorded in male alert Sprague Dawley rats after a recovery period of at least 24 hours, with a PowerLab/Labchart system (ADInstruments, Dunedin, New Zealand) connected to the arterial catheter via a pressure transducer. Mean arterial BP (MABP) was calculated from the arterial pressure signal as previously described [7]. The compound was administered s.c. after baseline BP recording for at least 30 minutes. BP was then measured at 3 and 24 hours after administration. At the end of the experiment, animals were killed with a lethal dose of pentobarbital (Dolethal ®, 150 mg/kg).

Supplementary references

1. Gerbier, R. *et al.* New structural insights into the apelin receptor: identification of key residues for apelin binding. *FASEB J.* **29**, 314–322 (2015).
2. Hus-Citharel, A. *et al.* Apelin Counteracts Vasopressin-Induced Water Reabsorption via Cross Talk Between Apelin and Vasopressin Receptor Signaling Pathways in the Rat Collecting Duct. *Endocrinology* **155**, 4483–4493 (2014).
3. Murza, A. *et al.* C-Terminal modifications of apelin-13 significantly change ligand binding, receptor signaling, and hypotensive action. *J. Med. Chem.* **58**, 2431–2440 (2015).
4. Zhang, Y. *et al.* Identifying structural determinants of potency for analogs of apelin-13: integration of C-terminal truncation with structure-activity. *Bioorg. Med. Chem.* **22**, 2992–2997 (2014).
5. De Mota, N. *et al.* Apelin, a potent diuretic neuropeptide counteracting vasopressin actions through inhibition of vasopressin neuron activity and vasopressin release. *Proc. Natl. Acad. Sci. U.S.A.* **101**, 10464–10469 (2004).
6. Azizi, M. *et al.* Reciprocal regulation of plasma apelin and vasopressin by osmotic stimuli. *J. Am. Soc. Nephrol.* **19**, 1015–1024 (2008).
7. Reaux, A. *et al.* Aminopeptidase A inhibitors as potential central antihypertensive agents. *Proc Natl Acad Sci U S A* **96**, 13415–13420 (1999).
