# Supplementary material for: A metabolically stable apelin-17 analog decreases AVP-induced antidiuresis and improves hyponatremia
Source: Nat Commun. 2021 Jan 12;12:305. doi: 10.1038/s41467-020-20560-y (PMC7804859; doi:10.1038/s41467-020-20560-y)
Supplement: Supplementary file 2 — Reporting Summary [file 41467_2020_20560_MOESM2_ESM.pdf]

nature research

Corresponding author(s): Catherine Llorens-Cortès

Last updated by author(s): Nov 14, 2020

Reporting Summary

Nature Research wishes to improve the reproducibility of the work that we publish. This form provides structure for consistency and transparency in reporting. For further information on Nature Research policies, see Authors & Referees and the Editorial Policy Checklist .

Statistics

For all statistical analyses, confirm that the following items are present in the figure legend, table legend, main text, or Methods section.

n/a	Confirmed	
☐	☒	The exact sample size (*n*) for each experimental group/condition, given as a discrete number and unit of measurement
☐	☒	A statement on whether measurements were taken from distinct samples or whether the same sample was measured repeatedly
☐	☒	The statistical test(s) used AND whether they are one- or two-sided<br>*Only common tests should be described solely by name; describe more complex techniques in the Methods section.*
☒	☐	A description of all covariates tested
☐	☒	A description of any assumptions or corrections, such as tests of normality and adjustment for multiple comparisons
☐	☒	A full description of the statistical parameters including central tendency (e.g. means) or other basic estimates (e.g. regression coefficient) AND variation (e.g. standard deviation) or associated estimates of uncertainty (e.g. confidence intervals)
☐	☒	For null hypothesis testing, the test statistic (e.g. *F*, *t*, *r*) with confidence intervals, effect sizes, degrees of freedom and *P* value noted<br>*Give P values as exact values whenever suitable.*
☒	☐	For Bayesian analysis, information on the choice of priors and Markov chain Monte Carlo settings
☒	☐	For hierarchical and complex designs, identification of the appropriate level for tests and full reporting of outcomes
☒	☐	Estimates of effect sizes (e.g. Cohen's *d*, Pearson's *r*), indicating how they were calculated

Our web collection on statistics for biologists contains articles on many of the points above.

Software and code

Policy information about availability of computer code

Data collection	Microsoft Excel for MacOS version 16
Data analysis	R version 3.6.3

For manuscripts utilizing custom algorithms or software that are central to the research but not yet described in published literature, software must be made available to editors/reviewers. We strongly encourage code deposition in a community repository (e.g. GitHub). See the Nature Research guidelines for submitting code & software for further information.

Data

Policy information about availability of data

All manuscripts must include a data availability statement. This statement should provide the following information, where applicable:

- Accession codes, unique identifiers, or web links for publicly available datasets
- A list of figures that have associated raw data
- A description of any restrictions on data availability

Source data are provided with this paper in a supplemental file.

Field-specific reporting

Please select the one below that is the best fit for your research. If you are not sure, read the appropriate sections before making your selection.

☒ Life sciences ☐ Behavioural & social sciences ☐ Ecological, evolutionary & environmental sciences

For a reference copy of the document with all sections, see nature.com/documents/nr-reporting-summary-flat.pdf

Life sciences study design

All studies must disclose on these points even when the disclosure is negative.

Sample size	For in vitro and ex vivo experiments, 3 to 5 seperate experiments were conducted to assess reproducibility.<br>For in vivo experiments, a total number of at least 5 animals per group was obtained. All parameters could not be obtained in the same experiments, while others (such as urine output) were systematically obtained. All data obtained during the study is presented in the manuscript (except for pre-defined exclusion criteria in figures 5 and S7), for exhaustivity of reporting, resulting in sometimes unbalanced groups.<br>For AVP-induced hyponatremia (Fig 5 and S7), sample size was determined before the study, based on previous results from the literature. For the hyponatremia study, the detection of a mean difference in plasma sodium concentration of 5 mmol/L (expected standard deviation of 3 mmol/L) between the treatment group and the control group would require the inclusion of at least 8 animals in each treatment group.
Data exclusions	For Figure 5 and Supplementary Figure S7, AVP-treated animals with an insufficient antidiuretic response (urinary osmolality < 850 mOsm/kg) were excluded. This cutoff was based from previous reports from the litterature and predefined before conducting the experiments.
Replication	Each in vitro experiment was performed at least three times in triplicate. For in vivo experiments, the presented results are obtained from at least 3 different experiments, conducted at different times using the exact same protocol, which each comprised the different experimental groups. Small number of animal for each treatment group in a given experiment did not allow to make separate statistical analysis, but a similar tendency was obtained in each experiment. The statistical analysis was performed on all experiments combined.
Randomization	All animals were randomly assigned to treatment groups.
Blinding	Blinding was not feasible for treatment administration and sample collection. Indeed, the compounds used (saline, LIT01-196 and tolvatpan) had a different macroscopic aspect and induced rapid and different physiological response (urine output, water intake) according to groups. However, the investigators were not aware of the treatment group during sample analysis (urine osmolality, plasma sodium determinations).

Behavioural & social sciences study design

All studies must disclose on these points even when the disclosure is negative.

Study description	*Briefly describe the study type including whether data are quantitative, qualitative, or mixed-methods (e.g. qualitative cross-sectional, quantitative experimental, mixed-methods case study).*
Research sample	*State the research sample (e.g. Harvard university undergraduates, villagers in rural India) and provide relevant demographic information (e.g. age, sex) and indicate whether the sample is representative. Provide a rationale for the study sample chosen. For studies involving existing datasets, please describe the dataset and source.*
Sampling strategy	*Describe the sampling procedure (e.g. random, snowball, stratified, convenience). Describe the statistical methods that were used to predetermine sample size OR if no sample-size calculation was performed, describe how sample sizes were chosen and provide a rationale for why these sample sizes are sufficient. For qualitative data, please indicate whether data saturation was considered, and what criteria were used to decide that no further sampling was needed.*
Data collection	*Provide details about the data collection procedure, including the instruments or devices used to record the data (e.g. pen and paper, computer, eye tracker, video or audio equipment) whether anyone was present besides the participant(s) and the researcher, and whether the researcher was blind to experimental condition and/or the study hypothesis during data collection.*
Timing	*Indicate the start and stop dates of data collection. If there is a gap between collection periods, state the dates for each sample cohort.*
Data exclusions	*If no data were excluded from the analyses, state so OR if data were excluded, provide the exact number of exclusions and the rationale behind them, indicating whether exclusion criteria were pre-established.*
Non-participation	*State how many participants dropped out/declined participation and the reason(s) given OR provide response rate OR state that no participants dropped out/declined participation.*
Randomization	*If participants were not allocated into experimental groups, state so OR describe how participants were allocated to groups, and if allocation was not random, describe how covariates were controlled.*

Ecological, evolutionary & environmental sciences study design

All studies must disclose on these points even when the disclosure is negative.

Study description	*Briefly describe the study. For quantitative data include treatment factors and interactions, design structure (e.g. factorial, nested, hierarchical), nature and number of experimental units and replicates.*
Research sample	*Describe the research sample (e.g. a group of tagged Passer domesticus, all Stenocereus thurberi within Organ Pipe Cactus National Monument), and provide a rationale for the sample choice. When relevant, describe the organism taxa, source, sex, age range and*

	any manipulations. State what population the sample is meant to represent when applicable. For studies involving existing datasets, describe the data and its source.
Sampling strategy	*Note the sampling procedure. Describe the statistical methods that were used to predetermine sample size OR if no sample-size calculation was performed, describe how sample sizes were chosen and provide a rationale for why these sample sizes are sufficient.*
Data collection	*Describe the data collection procedure, including who recorded the data and how.*
Timing and spatial scale	*Indicate the start and stop dates of data collection, noting the frequency and periodicity of sampling and providing a rationale for these choices. If there is a gap between collection periods, state the dates for each sample cohort. Specify the spatial scale from which the data are taken*
Data exclusions	*If no data were excluded from the analyses, state so OR if data were excluded, describe the exclusions and the rationale behind them, indicating whether exclusion criteria were pre-established.*
Reproducibility	*Describe the measures taken to verify the reproducibility of experimental findings. For each experiment, note whether any attempts to repeat the experiment failed OR state that all attempts to repeat the experiment were successful.*
Randomization	*Describe how samples/organisms/participants were allocated into groups. If allocation was not random, describe how covariates were controlled. If this is not relevant to your study, explain why.*
Blinding	*Describe the extent of blinding used during data acquisition and analysis. If blinding was not possible, describe why OR explain why blinding was not relevant to your study.*

Did the study involve field work? ☐ Yes ☐ No

Field work, collection and transport

Field conditions	*Describe the study conditions for field work, providing relevant parameters (e.g. temperature, rainfall).*
Location	*State the location of the sampling or experiment, providing relevant parameters (e.g. latitude and longitude, elevation, water depth).*
Access and import/export	*Describe the efforts you have made to access habitats and to collect and import/export your samples in a responsible manner and in compliance with local, national and international laws, noting any permits that were obtained (give the name of the issuing authority, the date of issue, and any identifying information).*
Disturbance	*Describe any disturbance caused by the study and how it was minimized.*

Reporting for specific materials, systems and methods

We require information from authors about some types of materials, experimental systems and methods used in many studies. Here, indicate whether each material, system or method listed is relevant to your study. If you are not sure if a list item applies to your research, read the appropriate section before selecting a response.

Materials & experimental systems

n/a	Involved in the study	
☐	☒	Antibodies
☐	☒	Eukaryotic cell lines
☒	☐	Palaeontology
☐	☒	Animals and other organisms
☒	☐	Human research participants
☒	☐	Clinical data

Methods

n/a	Involved in the study	
☒	☐	ChIP-seq
☒	☐	Flow cytometry
☒	☐	MRI-based neuroimaging

Antibodies

Antibodies used	AVP-[Arg8] antibody (dilution, 1/4.5) obtained from Peninsula Laboratories International (San Carlo, CA, USA), Cat# T-4561.<br>Mouse monoclonal anti-occludin antibody (dilution, 1/200) was obtained from Santa Cruz, Cliniscience, France (Cat# sc-133256).<br>Rabbit anti-AQP-2-pS269 antibody (dilution, 1/200) was obtained from Phospho Solution, Cliniscience, France. (Cat# p112-269).<br>Polyclonal K17F antiserum (rabbit polyclonal antibodies directed against the apelin fragment K17F) were produced in the laboratory, as previously described, and used with a 1/4500 dilution (ref 26, Reaux et al J. Neurochem 2001).
Validation	AVP-[Arg8] antibody: http://penlabs.com/wp-content/uploads/2014/06/T-4561-_Arg8_-Vasopressin-RAS1.pdf<br>Mouse monoclonal anti-occludin antibody Cat# sc-133256 : https://datasheets.scbt.com/sc-133256.pdf<br>Rabbit anti-AQP-2-pS269 Cat# p112-269 : https://www.phosphosolutions.com/wp-content/uploads/2015/11/p112-269-datasheet-8.pdf<br>Polyclonal K17F antiserum:

- Characterization of the K17F Antiserum. Mean IC50 values for binding inhibition of [125I]pE13F to the apelin antiserum was 0.28 ± 0.08 nM. Minimal concentration of pE13F necessary to significantly displace the tracer was 6 fmol. Crossreactivity of the apelin antiserum with various N- and C-terminally truncated fragments of K17F and several other bioactive peptides is as follows: taking reactivity with pE13F as 100%, the rank order of reactivity was K17F > pE13F = L36F ~ R12F > P11F > R10F > G5F >> K16P = K15M, with negligible reactivity observed for AngII, AngIII, neuropeptide Y, and AVP (De Mota et al, PNAS, 2004, in our manuscript ref n°20).

- Validation of the K17F Antiserum. To define the molecular forms of apelin produced in vivo, and detected by our antibody:
o in rats, we combined separation by gel permeation chromatography and RIA detection and identified pE13F and, to a lesser extent, K17F as the predominant forms of apelin present in the whole brain, hypothalamus, and plasma (De Mota et al, PNAS, 2004, in our manuscript ref n°20).
o In human plasma, by combining HPLC analysis with RIA detection, we showed that K17F and pE13F are the predominant forms of apelin present in human plasma and that the concentration of apelin 36 is much lower (Azizi et al. PNAS, 2008, in our manuscript ref n°21)

Eukaryotic cell lines

Policy information about cell lines

Cell line source(s)	CHO-K1 cells were obtained from American Type Culture Collection, Rockville, MD, USA.<br><br>HEK-293T cells were obtained from Cancer Research UK, London Research institute.<br><br>mpkCCDcl4 cell line was provided by Dr Michel-Robert Popoff from the Pasteur Institute, Paris, France. The original source of these cells is reported in the following reference mentioned in our manuscript reference n°49:<br><br>Bens M, Vallet V, Cluzeaud F, Pascual-Letallec L, Kahn A, Rafestin-Oblin ME, Rossier BC, Vandewalle A. Corticosteroid-dependent sodium transport in a novel immortalized mouse collecting duct principal cell line. J Am Soc Nephrol. 1999 May;10 (5):923-34. PMID: 10232677
Authentication	None of the cell lines used were authenticated
Mycoplasma contamination	Cell lines were tested negative for mycoplasma contamination
Commonly misidentified lines<br>(See ICLAC register)	None

Palaeontology

Specimen provenance	*Provide provenance information for specimens and describe permits that were obtained for the work (including the name of the issuing authority, the date of issue, and any identifying information).*
Specimen deposition	*Indicate where the specimens have been deposited to permit free access by other researchers.*
Dating methods	*If new dates are provided, describe how they were obtained (e.g. collection, storage, sample pretreatment and measurement), where they were obtained (i.e. lab name), the calibration program and the protocol for quality assurance OR state that no new dates are provided.*

☐ Tick this box to confirm that the raw and calibrated dates are available in the paper or in Supplementary Information.

Animals and other organisms

Policy information about studies involving animals; ARRIVE guidelines recommended for reporting animal research

Laboratory animals	Male swiss mice (25-30 g, 4-6 weeks) and male Sprague Dawley rats (210-230g, 6-8 weeks) were obtained from Charles River Laboratories (L'Arbresle, France). Animals were housed in our animal facility, with 12h light/dark cycles. Temperature was maintained between 18 and 22°C, and humidity between 40 and 60%.
Wild animals	No wild animals were used in the study.
Field-collected samples	No field collected samples were used in the study.
Ethics oversight	The experimental protocols were approved by the national animal ethics committee (CEEA, reference numbers 2016-10#3672, 2017-01 #7844 and 01966.02) and ethics regional committee for animal experimentation in Strasbourg (APAFIS reference number 1341#2015080309399690).

Note that full information on the approval of the study protocol must also be provided in the manuscript.

Human research participants

Policy information about studies involving human research participants

Population characteristics	*Describe the covariate-relevant population characteristics of the human research participants (e.g. age, gender, genotypic information, past and current diagnosis and treatment categories). If you filled out the behavioural & social sciences study design questions and have nothing to add here, write "See above."*
Recruitment	*Describe how participants were recruited. Outline any potential self-selection bias or other biases that may be present and how these are likely to impact results.*
Ethics oversight	*Identify the organization(s) that approved the study protocol.*

Note that full information on the approval of the study protocol must also be provided in the manuscript.

Clinical data

Policy information about clinical studies

All manuscripts should comply with the ICMJE guidelines for publication of clinical research and a completed CONSORT checklist must be included with all submissions.

Clinical trial registration	*Provide the trial registration number from ClinicalTrials.gov or an equivalent agency.*
Study protocol	*Note where the full trial protocol can be accessed OR if not available, explain why.*
Data collection	*Describe the settings and locales of data collection, noting the time periods of recruitment and data collection.*
Outcomes	*Describe how you pre-defined primary and secondary outcome measures and how you assessed these measures.*
